# Advances in understanding of the pathogenesis and therapeutic implications of drug reaction with eosinophilia and systemic symptoms: an updated review

**DOI:** 10.3389/fmed.2023.1187937

**Published:** 2023-06-29

**Authors:** Chun-Bing Chen, Wei-Kai Hung, Chuang-Wei Wang, Chih-Chun Lee, Shuen-Iu Hung, Wen-Hung Chung

**Affiliations:** ^1^Department of Dermatology, Drug Hypersensitivity Clinical and Research Center, Chang Gung Memorial Hospital, Linkou, Taiwan; ^2^Cancer Vaccine and Immune Cell Therapy Core Laboratory, Department of Medical Research, Chang Gung Memorial Hospital, Linkou, Taiwan; ^3^Chang Gung Immunology Consortium, Chang Gung Memorial Hospital and Chang Gung University, Taoyuan, Taiwan; ^4^Department of Dermatology, Xiamen Chang Gung Hospital, Xiamen, China; ^5^Xiamen Chang Gung Allergology Consortium, Xiamen Chang Gung Hospital, Xiamen, China; ^6^School of Medicine, National Tsing Hua University, Hsinchu, Taiwan; ^7^College of Medicine, Chang Gung University, Taoyuan, Taiwan; ^8^Graduate Institute of Clinical Medical Sciences, College of Medicine, Chang Gung University, Taoyuan, Taiwan; ^9^Immune-Oncology Center of Excellence, Chang Gung Memorial Hospital, Linkou, Taiwan; ^10^Department of Medical Education, Chang Gung Memorial Hospital, Keelung, Taiwan; ^11^Whole-Genome Research Core Laboratory of Human Diseases, Chang Gung Memorial Hospital, Keelung, Taiwan; ^12^Department of Dermatology, Beijing Tsinghua Chang Gung Hospital, School of Clinical Medicine, Tsinghua University, Beijing, China; ^13^Department of Dermatology, Ruijin Hospital, School of Medicine, Shanghai Jiao Tong University, Shanghai, China; ^14^Genomic Medicine Core Laboratory, Chang Gung Memorial Hospital, Linkou, Taiwan

**Keywords:** drug reaction with eosinophilia and systemic symptoms, DIHS, hypersensitivity, eosinophilia, HHV-6

## Abstract

Drug reaction with eosinophilia and systemic symptoms or drug-induced hypersensitivity syndrome (DRESS/DIHS) is one type of severe cutaneous adverse reaction (SCAR). It is featured by fever, widespread skin lesions, protracted clinical course, internal organ involvement, and possibly long-term autoimmune sequelae. The presence of high-risk human leukocyte antigen (HLA) alleles, hypersensitivity reaction after culprit drug ingestion, and human herpesvirus reactivation may all contribute to its complex clinical manifestations. Some recent studies focusing on the roles of involved cytokines/chemokines and T cells co-signaling pathways in DRESS/DIHS were conducted. In addition, some predictors of disease severity and prognosis were also reported. In this review, we provided an update on the current understanding of the pathogenesis, potential biomarkers, and the relevant therapeutic rationales of DRESS/DIHS.

## 1. Introduction

Drug hypersensitivity reaction are adverse effects that occur when the immune system reacts inappropriately to a medication. These reactions can range from mild to severe and can involve various organs and tissues, with the skin being the most commonly affected. These reactions can be categorized into two primary types: immediate drug hypersensitivity reactions and delayed drug hypersensitivity reactions (1, 2). Immediate hypersensitivity reactions occur within hours of drug exposure and are typically mediated by Immunoglobulin E (IgE) antibodies. These reactions can manifest as urticaria (hives), angioedema (swelling of the deeper layers of the skin), bronchospasm, or anaphylaxis. Delayed hypersensitivity reactions, on the other hand, occur days to weeks after drug exposure and are usually mediated by T cells. These reactions can present as various skin manifestations, including maculopapular eruptions, or severe cutaneous adverse reactions (SCARs), such as Stevens–Johnson syndrome and toxic epidermal necrolysis (SJS/TEN), acute generalized exanthematous pustulosis, and drug reaction with eosinophilia and systemic symptoms (DRESS). These severe reactions can cause systemic involvements and can be fatal (1, 2). DRESS syndrome is one of the life-threatening drug-induced SCARs. It is characterized by distinctive cutaneous manifestations, internal organ involvement, hematologic abnormalities, and probably long-term autoimmune sequelae (3). Typically, the symptoms develop 2 weeks to 3 months after taking the causative drugs. Patients may present with extensive erythema, facial edema, lymphadenopathy, and high-grade fever (4). Various degrees of internal organ involvement including hepatitis, nephritis, interstitial pneumonia, and myocarditis may occur. Marked elevated levels of blood eosinophils and the presence of atypical lymphocytes are hallmarks of hematologic manifestations.

Historically, similar manifestations were described as a variety of entities according to the causative drugs, like phenytoin hypersensitivity (5), dapsone hypersensitivity (or sulfone syndrome) (6), and allopurinol hypersensitivity syndrome (7). The term “DRESS” was first introduced by Bocquet et al. to make the description of this syndrome more consistent and unambiguous (8). Instead, another term “drug-induced hypersensitivity syndrome (DIHS)” is more widely used by Japanese experts (9). The diagnostic criteria of DRESS defined by the Registry of Severe Cutaneous Adverse Reactions (RegiSCAR) group and the criteria of DIHS are similar except for the inclusion of the status of Human herpesvirus 6 (HHV-6) reactivation in the diagnostic criteria of DIHS (10, 11). Current consensus denotes that these two terms (DRESS and DIHS) are likely within the same disease spectrum, and the diagnosis of DIHS may represent a more severe phenotype (10). In this review, we aimed to summarize the updated understanding of the pathogenesis and biomarkers of DRESS/DIHS as well as the relevant therapeutic implications.

## 2. Pathogenesis in drug reaction with eosinophilia and systemic symptoms

**Figure 1:**
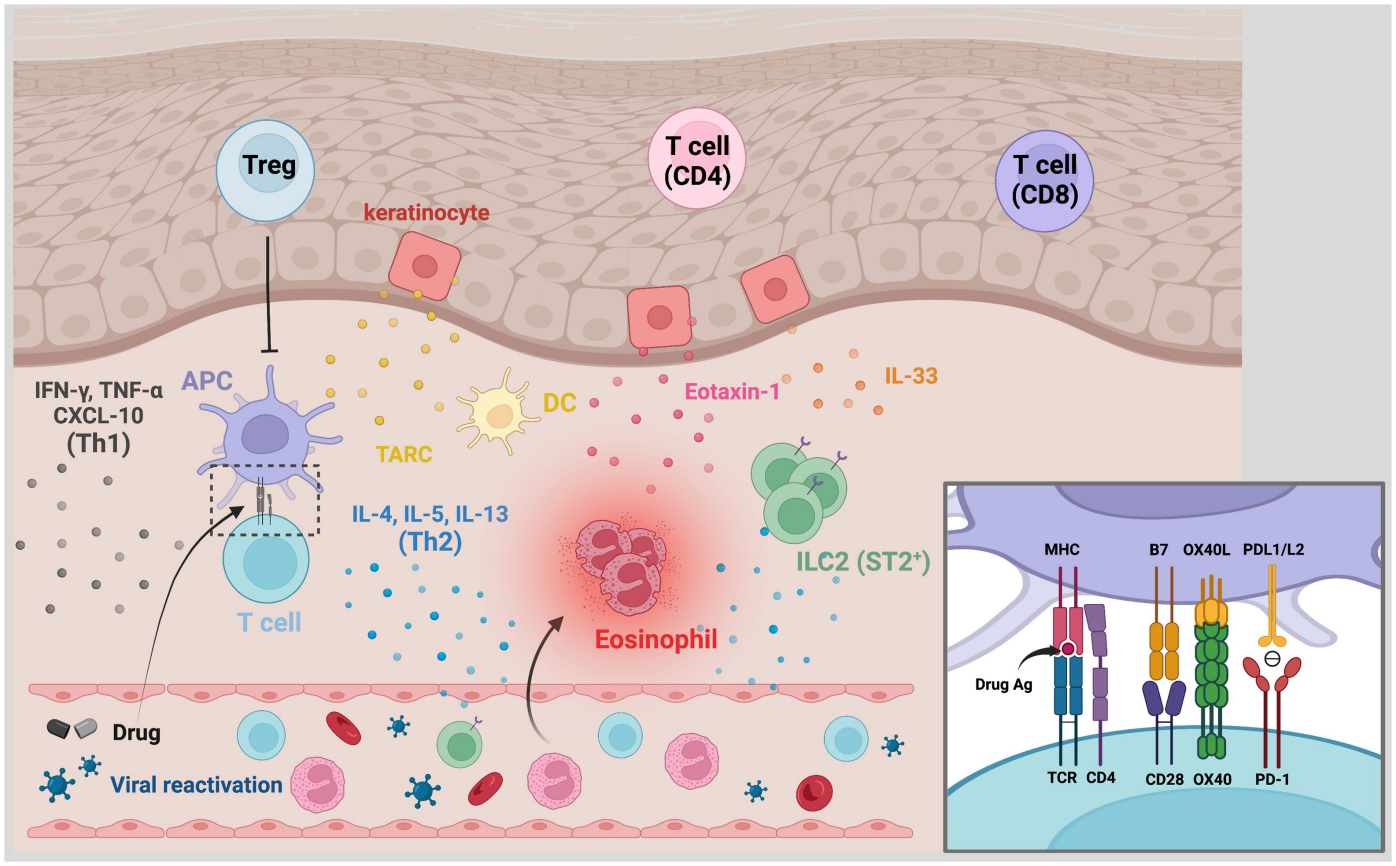
Pathogenesis of DRESS/DIHS. The affected skin is rich in CD4^+^ and CD8^+^ T cells, regulatory T cells (Tregs), plasma dendritic cells, and type 2 innate lymphoid cells (ILC2). TARC secreted by keratinocytes and DCs would recruit type 2 T helper (Th2) cells to the skin. Through antigen presentation, drug-specific T cells are activated and produced Th2-associated cytokines including IL-4, IL-5, and IL-13. Increased IL-33 levels, which may be secreted by keratinocytes and macrophages, activate ILC2 via its receptor ST2 and promote the production of Th2-associated cytokines. The IL-5, eotaxin-1, and TARC synergistically promote the local accumulation of harmful eosinophils. Th1-associated cytokines and chemokines like IFN-γ, TNF-α, and C-X-C motif chemokine 10 (CXCL-10) have also overexpressed in some cases. Viral reactivation is another characteristic hallmark in DRESS/DIHS. The inset shows the involved signals of antigen presentation. Traditionally, the specific antigen presented by the MHC molecule is recognized by antigen-specific TCR with the aid of B7-CD28 costimulation. The costimulatory interaction between OX40L and OX40, which is presented by activated T cells, may prevent T cells from being inhibited by Tregs. The binding of PD-1 and PDL1/L2 otherwise transmit signals to inhibit T cell proliferation and cytokines production. A compromised inhibitory mechanism of Tregs and PD-1/PDL1/L2 axes can lead to the promotion of these hypersensitivity reactions. CXCL-10, C-X-C motif chemokine 10; DC, dendritic cell; DRESS/DIHS, drug reaction with eosinophilia and systemic symptoms/drug-induced hypersensitivity syndrome; IFN-γ, interferon-γ; ILC2, type 2 innate lymphoid cells; PD-1, programmed cell death protein-1; PD-L1, programmed death ligand 1; TARC, thymus activation-regulated chemokine; TNF-α, tumor necrosis factor-α; Treg, regulatory T cell.

DRESS is considered a T cell-mediated delayed-type hypersensitivity reaction in the Gell and Coombs classification (12). Traditionally, DRESS is classified as a type IVb reaction that corresponds with CD8^+^ and CD4^+^ T cells responses underlying the production of interferon-γ (IFN-γ), interleukin (IL)-4, IL-5, and IL-13, resulting in eosinophilia. Currently, there are several proposed pathomechanisms majorly involved in DRESS. DRESS is a drug hypersensitivity reaction due to specific culprit drugs induced immune response in genetically susceptible patients, and HHV reactivation may synergistically contribute to its pathogenesis. The diverse manifestations of DRESS/DIHS may result from the complex interplay between the drug-specific and the antiviral immune responses (Figure 1).

### 2.1. Genetic susceptibility

Human leukocyte antigen (HLA) molecules have an essential role in the immune reaction. They specifically present antigens to the T cell receptors (TCRs) (13). Antigen-specific T cells are then selected and the following immune responses are initiated (13). The HLA genes are located at the major histocompatibility complex (MHC) region on chromosome 6p21.3 and are the most polymorphic genes in the human genome (14, 15). Many pharmacogenetic studies have been conducted to investigate the association between HLA alleles and the specific drug-induced DRESS/DIHS. Until now, a variety of risky HLA alleles have been reported. These associated HLA alleles are usually drug- and ethnic-specific (see Table 1). The results suggest these “risky” HLA molecules may preferentially present specific drug antigens to specific TCRs and initiate adverse immune responses. Therefore, people with risky HLA alleles are more susceptible to specific drug hypersensitivity reactions. According to the different prevalence of specific risk alleles in different ethnicities, pre-prescription screening tests were recommended by some organizations for populations at risk (68). The preemptive strategies have demonstrated some success in lowering the incidence of SCARs (69).

**Table 1:** HLA alleles with risks in drug hypersensitivities in different populations.

	HLA allele	Phenotype	Ethnicity
**Aromatic anticonvulsants**			
All^†^	B*08:01, B*13:01, B*56:02	DRESS	Thai (16)
Carbamazepine	A*31:01	DRESS/SJS/TEN	Northern European (17), Japanese (18), Korean (19)
		DRESS	Han Chinese (20, 21), European (20, 22), Spanish (23)
	A*31, Cw*04	DRESS	Iranian (24)
	B*15:11^‡^	MPE/DRESS/SJS	Han Chinese (25)
Phenytoin	B*13:01	DRESS	Thai (26)
	B*51:01	DRESS	Thai (27), Thai children (28)
	B*15:13	DRESS/SJS/TEN	Malaysian (29)
	B*56:02/04	DRESS	Thai (26)
	CYP2C19*3	DRESS	Thai (26)
	CYP2C9*3	DRESS/SJS/TEN	Han Chinese, Japanese, Malaysian (30)
	A*24:02	DRESS	Spanish (23)
	C*14:02	DRESS	Thai children (28)
Lamotrigine	A*24:02	DRESS	Spanish (23)
	A*31:01	DRESS/SJS/TEN	Korean (31)
	A*68:01	HSS^§^/SJS/TEN	European (32)
Allopurinol	B*58:01	HSS^§^ DRESS/SJS/TEN	Han Chinese (33), Thai (34), Japanese (35), Korean (36)
	A*24:02, DRB1*13:02^††^	DRESS	Korean (37)
**Antiretroviral drugs**			
Abacavir	B*57:01	HSS^§^	Americans (38, 39), Australians (40), Whites and Hispanics (41), Indian children (42)
Nevirapine	DRB1*01:01	HSS^§^	Australian (43)
	B*35:05	HSS^§^	Thai (44)
	B*14:02, Cw*08:01, Cw*08:02	HSS^§^	Sardinian (45), Japanese (46)
	C*04:01	DRESS/SJS/TEN	Malawian (47)
	Cw*04	HSS^§^	Han Chinese (48)
Raltegravir	B*53:01	DRESS	African (49, 50)
**Antibiotics and anti-inflammatory drugs**			
Dapsone	B*13:01	HSS^§^	Han Chinese (51), Indonesian (52), Korean (53)
		DRESS	Han Chinese (54, 55), Malaysian (55), Thai (54)
Co-trimoxazole	B*13:01	DRESS/SJS/TEN	Han Chinese (56), Malaysian (56), Thai (56–58)
Salazosulfapyridine	B*13:01	DRESS	Han Chinese (59)
Sulfonamide	A*11:01	DRESS/SJS/TEN	Japanese (60)
Piperacillin/tazobactam	B62	DRESS	United Kingdom (61)
Vancomycin	A*32:01	DRESS	European Americans (62), Spanish (63), Han Chinese (64)
	B*07:05	DRESS	Han Chinese (64)
	B*40:06	DRESS	Han Chinese (64)
	B*67:01	DRESS	Han Chinese (64)
Others			
Benznidazole	A*68	MPE/DRESS	Latin American (65)
	A*11:01	MPE/DRESS	Latin American (65)
	A*29:02	MPE/DRESS	Latin American (65)
Azathioprine	C*06:02	HSS^§^	Australian (66)
IL-1/IL-6 inhibitors	DRB1*15	DRESS	European (67)

¶ The risk alleles both for MPE, DRESS, and SJS/TEN are listed but risk alleles only for SJS/TEN are not listed in this table.

§ Hypersensitivity syndrome (HSS) is a historical term that may not fulfill the current diagnostic criteria of DRESS but have similar clinical manifestations in common.

† Include phenytoin, carbamazepine, lamotrigine, phenobarbital, and oxcarbazepine.

‡ Only limited cases are reported (1 in SJS, 2 in DRESS, and 1 in MPE).

†† This study investigated Korean patient who are HLA-B*58:01 (+) carriers. The frequency of A*24:02/DRB1*13:02 was significantly higher in the B*58:01 (+) DRESS group than in the B*58:01 (+) tolerant controls.

DRESS, drug reaction with eosinophilia and systemic symptoms; HSS, hypersensitivity syndrome; MPE, maculopapular exanthema; SJS, Stevens-Johnson syndrome; TEN, toxic epidermal necrolysis.

### 2.2. Models of drug recognition by drug-specific T cells

**Figure 2:**
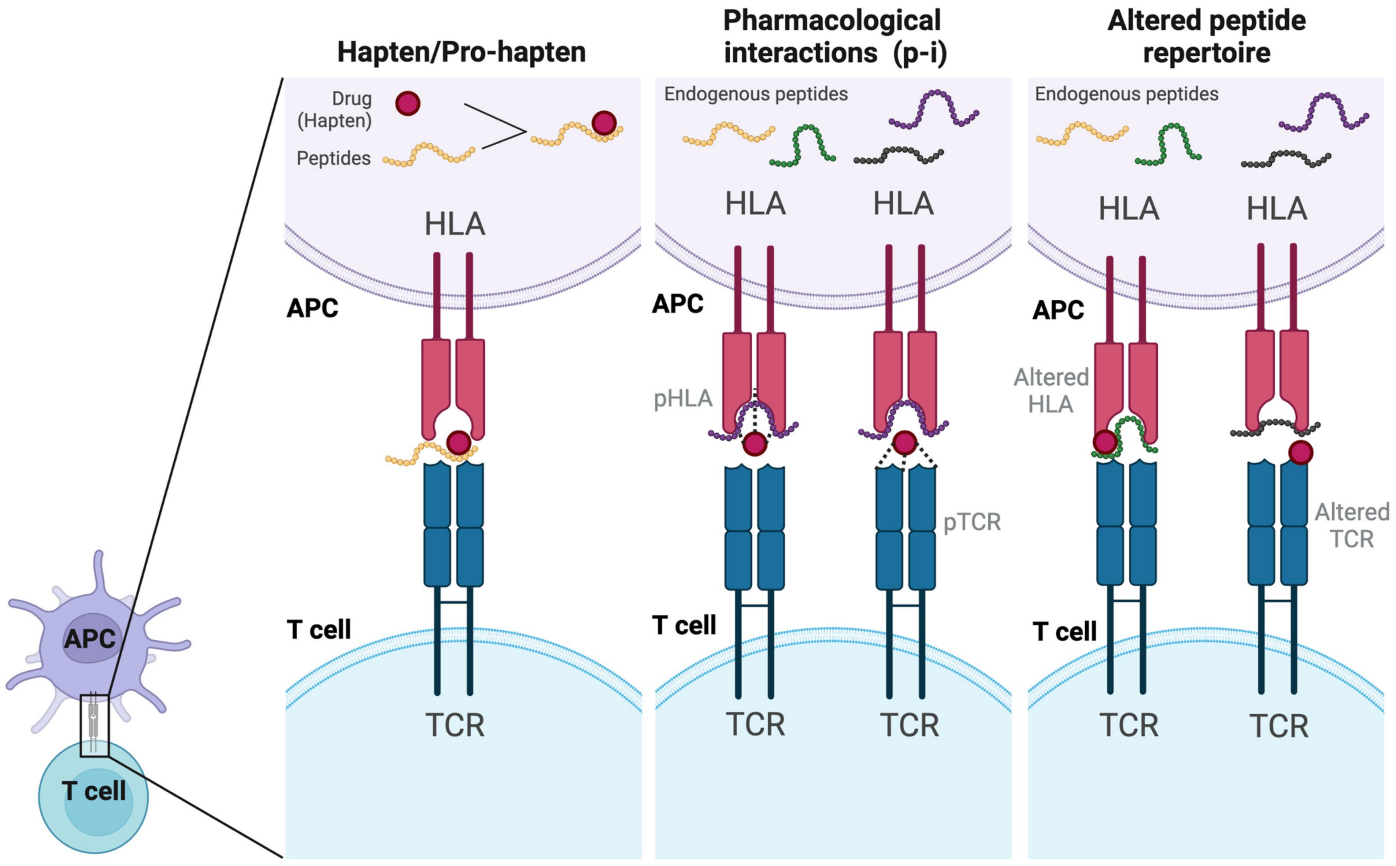
Models of antigen presentation and T-cell activation by drugs. In the hapten/pro-hapten model, the drug (hapten) covalently binds to a larger self-peptide and forms a neoantigen which could be presented by specific HLA alleles and recognized by the specific T cell receptor. In the p-i model, the drug would bind to the HLA or TCR noncovalently, which is a processing-independent interaction and can stimulate T cell activation directly. In the altered self-peptide repertoire model, the drug may bind to either the HLA (altered HLA) or TCR (altered TCR), leading to a conformational change and altering the repertoire of self-peptides presented to T cells. APC, antigen-presenting cell; HLA, human leukocyte antigen; TCR, T cell receptor.

Traditionally, it was commonly believed that T cells typically recognized peptides with at least 8–9 amino acids (MHC class I) and 12–15 amino acids (MHC class II) presented by antigen-presenting cells (APCs) (70). In drug hypersensitivity, several models were proposed for recognition of the small drug compounds by T cells with subsequent initiation of immune response. Currently, three main models were widely discussed (Figure 2): the hapten/pro-hapten model, the pharmacological interactions model (p-i concept), and the altered peptide repertoire model (71). In the hapten/pro-hapten model, the drug (hapten) or its reactive metabolites (pro-hapten after processing) would covalently bind to a larger protein or peptide. This formation of a hapten-protein/peptide complex called “haptenation” make this complex recognizable to the T cell receptor and gains the ability to activate the downstream immune response (71). The theory was best demonstrated in cases of penicillin-induced cutaneous adverse reactions (72). In the p-i concept, by contrast, the causative drugs may noncovalently and directly bind to the immune receptors like TCR or specific HLA molecules. The binding of these drugs is sufficient to stimulate the signaling transmission through the TCR without the need for a classic antigen-processing pathway (73). This concept was demonstrated and introduced by delicate studies conducted by Pichler’s group (74–76) and supported by the study conducted by Wei et al. (77) in the field of SCAR. In the study conducted by Wei et al. (77), the carbamazepine can directly interact with the HLA-B*15:02 molecule, be presented to APCs, and initiate immune responses (77). No intracellular antigen processing was involved in the HLA presentation of carbamazepine (77). Similar interaction was also seen in the hypersensitivity reaction induced by dapsone (78). The concept of the altered peptide repertoire model was demonstrated in the case of abacavir hypersensitivity syndrome in HLA-B*57:01-positive individuals (79–81). Abacavir binds noncovalently to the antigen-binding groove of HLA-B*57:01, which causes the conformational changes of the binding groove and alters the repertoire of endogenous peptides presented by HLA-B*57:01. Therefore, previously tolerated self-peptides become recognizable and may elicit hypersensitivity reactions. However, interestingly, these models may not be mutually exclusive in drug hypersensitivity reactions. For example, the hapten/pro-hapten theory and p-i concept had both been proposed in sulfamethoxazole-induced hypersensitivity reactions (82, 83). Different antigen-presenting mechanisms may be involved in different cases, further investigation may be needed to elucidate the actual mechanism in different drugs-induced DRESS/DIHS.

### 2.3. Costimulatory/coinhibitory signaling pathways

For T cell activation to be initiated, two signals are required: the interaction of TCR MHC/TCR interaction (signal 1), and simultaneous costimulatory signals (signal 2) provided by a set of costimulatory molecules. The interaction between CD28 presented on T cells and the CD80/CD86 expressed on the surface of APCs is the prototype for this crucial signaling pathway. Asides from the CD28-CD80/CD86, there are several costimulatory and coinhibitory signaling molecules playing potentially important roles in the pathogenesis of DRESS/DIHS, such as OX40/OX40L, PD-1/PD-L1, and CTLA-4/CD80/CD86 axis (84).

#### 2.3.1. OX40 (CD134) and OX40L

OX40, also known as CD134, is a member of the tumor necrosis factor (TNF) receptor superfamily. Unlike other molecules in the TNF receptor superfamily, the OX40 was not expressed by naïve T cells but transiently expressed by antigen-activated T cells. The ligand for OX40 (OX40L) is expressed broadly by professional APCs, vascular endothelial cells, activated natural killer (NK) cells, and the responding CD4 T cells (85). At least two mechanisms of OX40-OX40L interaction were suggested including (1) promoting the expansion and survival of effector T cells and the generation of memory T cells, and (2) disrupting T-cell tolerance by antagonizing Treg-mediated suppression (85). The pathogenic role of OX40-OX40L interaction has been demonstrated in various immune diseases and cancers (86, 87).

Beyond the above-mentioned role in T cell activation, recent studies further delineated another role of OX40 in DRESS/DIHS. Miyagawa et al. found both the OX40-expressing CD4+ T cells and the OX40L-positive cells (in peripheral blood mononuclear cells, PBMCs) were upregulated in DRESS/DIHS in the acute stage compared with other drug eruptions (88, 89). HHV-6 reactivation is regarded to be one of the key contributors to the development of DRESS/DIHS. To be noticed, OX40 was previously identified as a specific receptor that helps the entry of HHV-6 to T cells (90). Recent studies demonstrated that the levels of serum soluble OX40 (sOX40), which may be produced by OX40 shedding and/or alternative splicing, were also increased in the serum of DRESS/DIHS patients, and were correlated with the peak level of HHV-6 viral loads (91). Moreover, the increased percentage of OX40 expression and level of sOX40 were both correlated with the serum levels of TARC/CCL17, a Th2-associated chemokine that is associated with disease severity in patients of DRESS/DIHS (92). In another study, Lee et al. demonstrated increased OX40-expressing CD4+ T cells in the lesional skin in DRESS/DIHS, and the frequency of OX40+ CD4 T cells was also correlated with the DRESS/DIHS severity score (93). These advanced investigations suggest that OX40/OX40L axis plays essential roles not only in T cell activation and Treg regulation but also in the HHV-6 replication and initiation of DRESS/DIHS.

#### 2.3.2. Programmed death (PD)-1/programmed death-ligand-1/(PD-L1) and cytotoxic T-lymphocyte–associated antigen (CTLA)-4

PD-1, PD-L1, and CTLA-4 are well-known coinhibitory molecules since the concept of immune checkpoint blockade emerged as a promising strategy to defeat cancers. Many monoclonal antibodies targeting these molecules or their ligands were approved and have had a large success in cancer treatment (94).

PD-1 is a checkpoint receptor primarily found on activated CD4+ T cells, activated CD8+ T cells, and peripheral B cells (95). Its ligand PD-L1 is expressed on T cells, B cells, macrophages, and dendritic cells (DCs) while another ligand PD-L2 is expressed primarily on APCs (95). Ligation of PD-1/PD-L initiates immunosuppressive signals and inhibits the T cells proliferation, cytokine production, and cytotoxicity of T cells (95, 96). CTLA-4, a structural homolog of CD28, is expressed on CD4+ T cells and CD8+ T cells and would competitively bind to the same ligands of CD80/CD86 on APCs (87). The binding of CTLA-4 transmits an inhibitory signal to negatively regulate the T cell’s response (97).

There have been several case reports of immune checkpoint inhibitors induced DRESS/DIHS, showing the potential involvement of these coinhibitory molecules in the pathogenesis of DRESS/DIHS. Two cases developed DRESS/DIHS after using nivolumab, one is for metastatic renal cell carcinoma and another one is for gastric carcinoma (98, 99). For treating metastatic melanoma, two cases developed DRESS/DIHS after using ipilimumab alone or the combined use of ipilimumab plus nivolumab (100, 101). In addition, an increased incidence of hypersensitivity reaction was also observed when patients received sulfasalazine with concurrent immune checkpoint inhibitors (102). This phenomenon suggests that the immune regulatory pathway acts as another factor in determining drug susceptibility (103, 104). Hammond et al. also provided some evidence that checkpoint inhibition may reduce the threshold for drug-specific T-cell priming, which explain the tendency of drug hypersensitivity in these population (105). The exact roles of these coinhibitory molecules in the development of DRESS/DIHS are still under-investigated, and further studies are warranted.

### 2.4. Involved cytokines and chemokines

Different types of cytokines and chemokines are involved in the pathogenesis of DRESS/DIHS (summarized in Table 2). DRESS/DIHS is classically considered a Th2-driven reaction with hallmarks of activated lymphocytes and eosinophilia. Th2-associated cytokines such as interleukin (IL)-4, IL-5, and IL-13 were secreted by activated Th2 cells and have a crucial impact on the development of DRESS/DIHS (118, 119). Increased levels of IL-5 were found in the plasma of hypersensitivity syndrome and associated with the generation of eosinophilia in these patients. IL-5 is not only a primary growth factor for eosinophils but also a Th2 chemokine, which plays a key role in promoting the differentiation, survival, and migration of eosinophils (120). Eotaxin-1, a chemokine also known as cysteine cysteine ligand 11 (CCL11), was also identified to be able to regulate eosinophils recruitment and activation synergistically with IL-5 (121). Circulating IL-4- and IL-13-producing CD4 + T cells were significantly higher in patients with DRESS (119). Of note, IL-13-producing T cells were significantly dominant skin-homing CLA+ cells, and the proportions of circulating IL-13-producing cells were correlated with serum thymus activation-regulated chemokine (TARC) levels.

**Table 2:** Cytokines, chemokines, and cytotoxic molecules involved in DRESS/DIHS.

Molecules	Secreted cells	Function in ADRs	Clinical significance	Tissue with high expression	References
Granulysin	CD8^+^ T cellsNK cells	Induce cell apoptosis	-	Skin, serum	(106, 107)
Granzyme B	CD8^+^ T cellsNK cells	Induce cell apoptosis	Correlated with liver function impairment	Skin, serum	(108)
IFN-γ	Th1 cellsCD8^+^ T cellsNK cells	Induces MHC II molecules on monocytes and keratinocytesStimulate IgG and IgMInhibit Th2 immune responseActivate macrophages and NK cells	Higher levels in patients with severe visceral involvement	PBMC	(109–111)
IL-2	Activated T lymphocytes	Stimulate T-cell activation and regulate T-cell functionsActivate NK cells	Higher levels in patients with severe visceral involvement	PBMC	(109–111)
IL-4	Th2 cellsMast cellNK cellsKeratinocytes	Decrease the production of Th1 cellsThe proliferation of activated B-cell and mature T-cellSwitch antibody class to IgEUpregulate MHC II expression	-	PBMC	(109)
IL-5	Th2 cellMast cells	Promote the formation and differentiation of eosinophilWork with IL-3 and GM-CSF synergistically	-	PBMC	(109, 111)
IL-6	Monocytes/macrophagesCD4^+^ T cellsKeratinocytes	Activation, growth, and differentiation of T cellsMaturation of B cells and production of antibodyInduces acute-phase protein production	Indicator of HHV-6 reactivation	Serum	(112)
IL-13	Th2 cellsMast cellsKeratinocytes	Inhibits macrophage activationActivate B cells, induces IgE antibodyDecreases pro-inflammatory cytokines in keratinocytes and endothelial cells	-	PBMC	(109, 111)
IL-15	Monocyte/macrophagesDendritic cellsEpithelial cells	Activation and proliferation of T and NK cellB-cell growth and differentiationMaturation of dendritic cells.	Predict the development of CMV reactivation	Serum	(113)
IL-17	Th17 cells	Activates macrophages, fibroblasts, keratinocytes, and endothelial cellMediate proinflammatory responses	-	PBMC	(110)
IL-33	Epithelial cellsFibroblastsEndothelial cells	Induce the Th2 immune response through its receptor ST2 triggered by infections or allergens	Its “decoy” receptor sST2 was elevated in the acute stage and correlated with disease activity	Skin, serum^¶^	(114)
IP-10/CXCL10	MonocytesEndothelial cellsFibroblasts	Th1 cell priming and differentiationChemoattraction for monocytes/macrophages, T cells, NK cells, and dendritic cells	Indicator of HHV-6 reactivationPredict the development of long-term sequelae	Skin, plasma	(115)
Soluble Fas ligand	CD8^+^ T cellsKeratinocytes	Induce cell apoptosis	Correlated with liver function impairment	Skin, serum	(108)
TARC/CCL17	KeratinocytesDendritic cellsEndothelial cells	Th2 cell chemokine and ligand for CCR4Eosinophil chemoattractant	Correlated disease activityIndicator of HHV-6 reactivation	Skin, serum	(92, 116, 117)
TNF-α	Monocytes/MacrophagesCD4^+^ and CD8^+^ T cellsKeratinocytes	Regulate keratinocyte apoptosisActivates vascular endotheliumActivates macrophages andstimulates nitric oxide productionInduces fever and septic shock	Indicator of HHV-6 reactivationHigher levels in patients with severe visceral involvement	Skin, serum, PBMC	(108, 110, 112)

¶ Increased level in serum of steroid-delayed responders.

ADRs, adverse drug reactions; CMV, cytomegalovirus; GM-CSF, granulocyte/macrophage colony-stimulating factor; HHV-6, human herpesvirus-6; IFN-γ, interferon-γ; IL, interleukin; IP-10, interferon-γ-induced protein-10; MHC, major histocompatibility complex; NK cell, natural killer cell; PBMC, peripheral blood mononuclear cell; TARC, thymus activation-regulated chemokine; TNF-α, tumor necrosis factor-α.

TARC, known as CC chemokine ligand 17 (CCL17), was found to be increased in the serum in DRESS/DIHS patients (92, 116). TARC was produced mainly by DCs, Langerhans cells, and keratinocytes (122). Its main ligand, CC chemokine receptor type 4 (CCR4), is expressed predominantly by Th2-type T cells (122, 123). The TARC/CCL17 can serve as a recruiter for CCR4+ Th2 cells to the inflamed tissue and enhance the type 2 immune responses (122, 123). In DRESS/DIHS, the increased level of serum TARC/CCL17 was found, which is correlated with the blood eosinophil count (124). Moreover, the higher level of TARC/CCL17 in DRESS/DIHS patients at the acute stage was also correlated with the disease activity, and it may predict the presence of HHV-6 reactivation (92, 116).

In addition, IL-33, a member of the IL-1 cytokine superfamily, also activates immune cells and promotes Th2-associated cytokines production *via* selectively binding to its receptor ST2 (125). Recently, an increased number of type 2 innate lymphoid cells (ILC2s) expressing ST2 were identified in the skin and blood in patients with DRESS/DIHS at the acute stage and these patients presented with increased serum soluble ST2 levels (114). The study suggests the IL-33/ ST2 pathway and ILC2s are involved in the pathogenesis of DRESS/DIHS and more studies may be warranted to confirm this relationship.

Nevertheless, the different types of immune reactions may not be exclusive. In addition to interleukin (IL)-4, 5, and 13, which are predominant cytokines involved in Th2 response, tumor necrosis factor (TNF)-α and interferon (IFN)-γ (typically for Th1 response) are also found to be overexpressed in DRESS/DIHS in some studies (109, 112, 126) and the elevated levels of TNF-α and IL-6 preceded the HHV-6 infection (112). TNF-α and IFN-γ were primarily produced by the expanded population of activated CD8+ T cells (110). Paradoxically, in patients with DRESS/DIHS and HHV-6 reactivation, TNF-α and IFN-γ levels were lower during an early stage (127). Moreover, overexpression of granulysin, granzyme B, and soluble Fas ligand (sFasL) in the skin lesions and increased levels of them in the serum were also noticed in DRESS/DIHS (106–108). The elevated levels of sFasL and granzyme B were also found to be correlated with the elevation of liver enzymes (108). Granulysin and granzyme B are two cytotoxic molecules that were regarded as key mediators in the SJS/TEN (128, 129). sFasL were found elevated in SJS/TEN and could be an indicator for early diagnosis of SJS/TEN (130). These findings suggest that these cytotoxic proteins may also play important roles in the pathogenesis of cutaneous and liver manifestations in DRESS/DIHS. Recently, interferon-γ-induced protein (IP)-10/CXCL10, which is a major chemokine involved in the Th1 cell priming and differentiation, has also been reported to be associated with the development of long-term sequelae in DIHS/DRESS and DRESS with HHV-6 reactivation (127, 131). The detailed role of these Th1 cytokines in DRESS/DIHS is not clear, and further research is warranted for clarifying their function.

In another study including an analysis of 40 DRESS/DIHS patients, overexpression of IL-17 and IL-17E was demonstrated (110). In previous mice models, IL-17E could regulate Th2 immune response and result in an elevated level of eosinophils, IL-4, IL-5, eotaxin, and immunoglobulin E (132). These results suggest IL-17E may also play some role in amplifying the immune response in DRESS/DIHS. Other cytokines such as IL-2 and IL-15 were also found elevated in the PBMC and serum in DRESS/DIHS patients, respectively (109–111, 113). The IL-2 was crucial for the proliferation and activation of T cells, and the elevation of IL-15 seemed to be associated with the development of CMV reactivation in DRESS/DIHS (113, 133).

### 2.5. Herpesvirus reactivation and antiviral responses

Association between the reactivation of latent viruses of the HHV family and DRESS/DIHS has been well studied. The most well-known scenario is HHV-6 reactivation, which can be found in 43–100% of patients with DRESS/DIHS and was included in the diagnostic criteria for DIHS (127, 134, 135). The presence of HHV-6 reactivation may represent a more severe phenotype (135, 136). Other members of the HHV family like EBV, CMV, and HHV-7 were also reported to be involved in the pathogenesis of DRESS/DIHS (110, 137).

HHV-6 infects T cells and establishes life-long latency in humans (138). One study demonstrated that the HHV-6-positive monomyeloid precursors (CD11b^+^CD13^+^CD14^−^CD16^high^) would be recruited to the lesional skin in the acute stage of DRESS/DIHS (139). The responsible chemoattractant may be high-mobility group box (HMGB)-1, which is a member of the damage-associated molecular pattern molecule (DAMP) family (139). The HMGB-1 level has been found to increase in the blood and skin in patients with DRESS/DIHS (139). Subsequently, the recruited monomyeloid precursors may potentiate the transmission of HHV-6 to the skin-resident CD4+ T cells (139). The HHV-6 infection of the CD4+ T cells subset serves as an indispensable step for the replication and activation of the virus (138, 139). In the acute stage of DRESS/DIHS, higher expression of OX40 (CD134), an entry receptor of HHV-6 as aforementioned, by activated CD4+ T cells has also been found (88). Hence, these studies suggested that the skin might be the primary site where HHV-6 reactivation starts (88, 139).

There have been several proposed mechanisms eliciting HHV-6 reactivation during the development of DRESS/DIHS. One possible theory is the direct effect of culprit drugs or their metabolites on viral reactivation. In one *in vitro* study conducted by Mardivirin et al., HHV-6 replication was increased by amoxicillin in the MT4 cell line (140). Similar effects were observed in other *in vitro* studies of valproic acid, which enhanced both HHV-6 and CMV replications (141, 142). Another theory proposed is related to the alterations of cellular and cytokine profiles in the early stage of DRESS/DIHS. In the early stage, the numbers of B cells and serum gammaglobulin reduced and Treg cells expanded (143–146). Serum levels of pro-inflammatory cytokines and chemokines such as TNF-α, IFN-γ, IL-1, IL-2, and IL-6 have been found lower in DRESS/DIHS patients with HHV-6 reactivation than the patients without (127). In addition, the plasmacytoid dendritic cells (pDCs), which produced type 1 interferon (IFN-α/β) upon activation to help neighboring cells resist viral infection, have also been found to have decreased levels in the circulation in patients with DRESS/DIHS around the time of viral reactivation (147–150). These relatively immunocompromised conditions were supposed to be related to the viral reactivation in DRESS/DIHS. On the contrary, the number of pDCs increased in the dermis of lesional skin, suggesting that pDCs may be recruited from circulation to skin during HHV-6 reactivation and enhance antiviral response nearby (147). In addition, the levels of TARC/CCL17 are also significantly elevated at an acute stage in DRESS/DIHS, and their levels are even higher in patients with the presence of HHV-6 reactivation (116, 117). As aforementioned, TARC/CCL17 would recruit CCR4+ Th2 cells and promote subsequent Th2 responses (122, 123).

**Figure 3:**
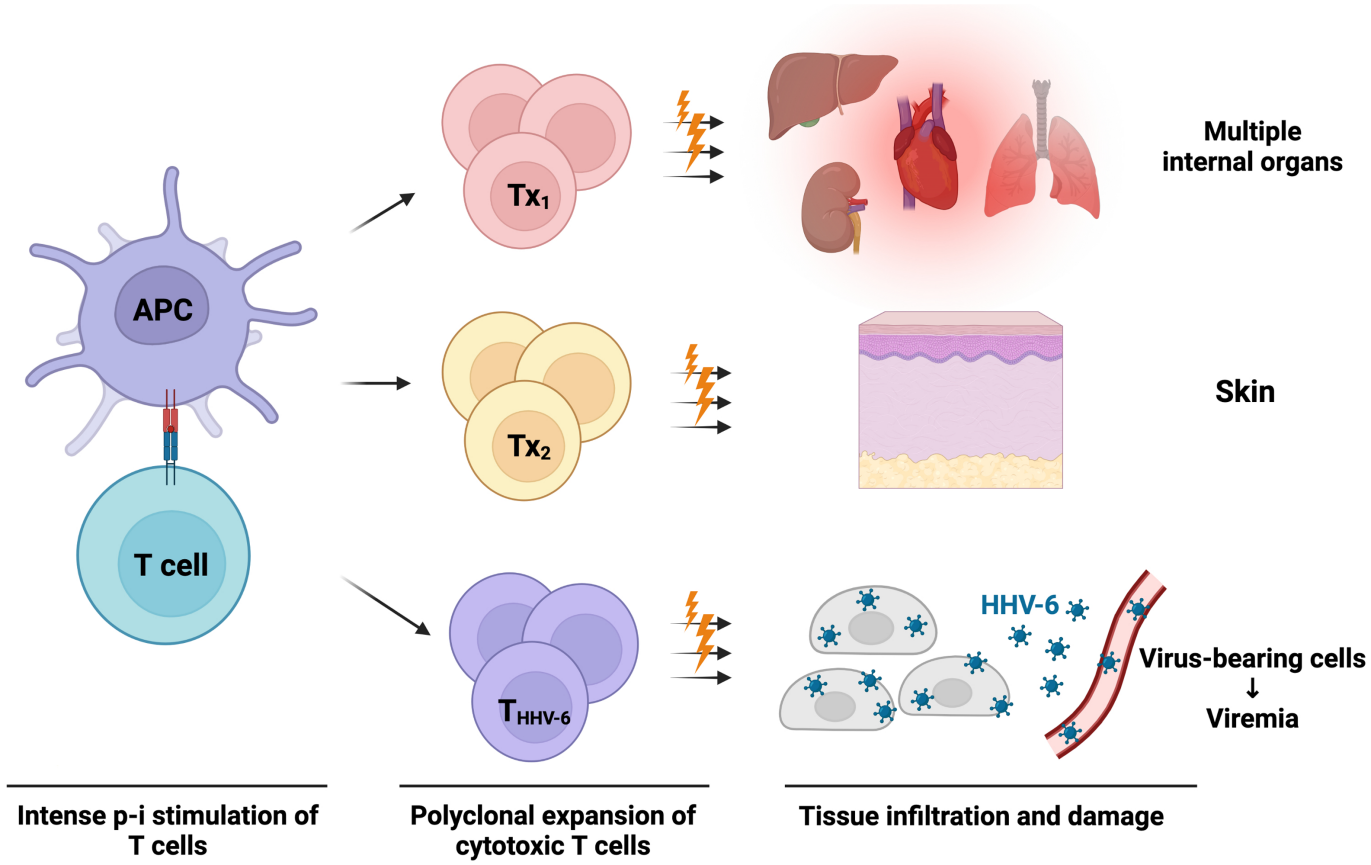
The virus-release hypothesis proposed by Pichler et al. (151) The massive p-i stimulation in DRESS/DIHS can lead to strong polyclonal T-cell expansion and activation. These p-i activated T cells are composed of cytotoxic T cells targeted at various internal organs, skin, and even virus peptide-specific T cells (may be reactive against HHV-6, CMV, EBV, etc.). These cytotoxic T cells attack organs, skin, and herpes peptide-expressing cells, causing acute symptoms of DRESS/DIHS and subsequent release of the prefabricated viruses. Therefore, viremia of various herpes viruses may be detected at a later stage. APC, antigen-presenting cell; CMV, cytomegalovirus; DRESS/DIHS, drug reaction with eosinophilia and systemic symptoms/drug-induced hypersensitivity syndrome; EBV, Epstein–Barr virus; HHV-6, human herpesvirus-6; T_HHV-6_, HHV-6 specific cytotoxic T cell; T_x_, drug-stimulated T cells against various tissue.

Recently, Pichler et al. postulated another theory called the “virus-release hypothesis” (151). In this hypothesis (Figure 3), DRESS/DIHS caused by intense drug-induced p-i stimulation would activate polyclonal cytotoxic T lymphocytes (151, 152). Within these p-i-activated T cells, there are also herpes virus-specific cytotoxic T cells. The virus-specific T cells kill the herpes virus-infected cells and lead to the release of intracellular herpes viruses. Therefore, blood viremia could be detected with or without symptoms of viral reactivation. This hypothesis can be supported by the previous study results, which denoted the possibility of simultaneously increased serum viral loads of different herpes viruses like HHV-6, CMV, and EBV (153). Most patients with viremia do not present with associated symptoms. However, patients with CMV reactivation are more commonly related to poor prognosis and complications (113, 151). Nevertheless, whether the herpes virus reactivation or viremia is a causative or reactive factor in the pathogenesis of DRESS/DIHS is still in debate. More studies are warranted to uncover this mysterious veil.

### 2.6. The COVID-19 era

The COVID-19 pandemic has adversely affected our healthcare system across different countries and has a large impact on dermatology practice (154, 155). Different kinds of dermatoses development after the prolonged use of self-protect equipment, COVID-19 infection, and administration of COVID-19 vaccines were frequently reported (155). Drug hypersensitivity reactions like maculopapular drug rashes, have been reported associated with COVID-19 infection (151, 156). There were several supporting evidence for the potential pathomechanism including an increased SARS-CoV-2 spike protein receptor (angiotensin-converting enzyme 2) expressed by keratinocytes, and SARS-CoV-2 RNA isolated from the skin of COVID-19 infected patients (156). The use of systemic treatment for these immune-mediated dermatological disorders could be more complex (157). Moreover, DRESS/DIHS and other SCARs were reported potentially to be induced by the COVID-19 vaccinations (158–160). Schroeder et al. reported a definite case of DRESS/DIHS after the administration of second dose of Pfizer/BioNtech COVID-19 vaccine (160). The authors hypothesized that the reaction of skin and internal organs may be caused by the inefficient detoxification of the vaccine and accumulation of the reactive metabolites (160). However, the exact immune mechanism and causal relationship between COVID-19 vaccines and SCARs are hard to ascertained. More research is warranted to delineate the pathogenesis of COVID-19 vaccines on SCARs.

## 3. Prognosis and potential biomarkers

DRESS/DIHS presents with diverse clinical manifestations and has potential life-threatening risks (3, 68, 161). Risk stratification and biomarker monitoring of these patients are important for guiding management strategy.

Clinically, the outcomes of patients may differ due to the different causative drugs. For example, anticonvulsants and allopurinol were reported to be associated with a poorer prognosis compared with other culprit drugs like antibiotics (162, 163). Dermatological features with the presence of facial edema (164), laboratory examinations with higher lymphocyte count (165), severe liver injury (165), and the presence of HHV reactivation (166) were also linked to a worse prognosis or more severe disease. In addition, tachycardia, leukocytosis, tachypnea, coagulopathy, gastrointestinal bleeding, and systemic inflammatory response syndrome were associated with poor outcomes in DRESS patients (167). Recently, there were two scoring systems proposed to assess the disease severity and predict outcomes. One scoring system proposed by Mizukawa et al. composed of multiple clinical and laboratory data, including age, duration of drug exposure, allopurinol exposure, pulsed steroid use, the extent of skin involvement, fever, appetite loss, renal dysfunction, liver dysfunction and elevated CRP (168). The patients with a total score ≥ 4 represent severe cases with a predilection for later development of CMV diseases and complications (168). The other risk prediction model developed by Sharma et al. included 6 variables (age, sex, rash morphology, facial edema, medication class, and antinuclear antibody positivity) that were associated with the risk of the recurrence of DRESS syndrome (169). However, these models are derived from relatively small numbers of cases and the generalizability to other populations needs further validation.

Aside from these clinical features, some biomarkers were mentioned in studies to provide diagnostic and prognostic clues. Serum TARC/CCL17, soluble ST2, and sOX40 levels were all elevated at the acute stage in patients with DRESS/DIHS (91, 114, 116, 124, 170). The serum TARC/CCL17 levels were correlated with other indicators of systemic inflammation, and the soluble ST2 levels were correlated with IL-33 and alanine aminotransferase levels (114, 170). These are potential biomarkers of early identification and disease severity stratification in DRESS/DIHS. In addition, the elevated levels of serum TNF-α, TARC/CCL17, and sOX40 were also possible indicators of HHV-6 reactivation in DRESS/DIHS (117, 170, 171). Developing a scoring system using the above distinct clinical presentations and potential biomarkers may be a promising strategy for risk stratification and better outcome prediction. However, the availability of measuring these biomarkers may limit the application, especially in resource-poor healthcare facilities.

## 4. Treatments

Since DRESS/DIHS is a relatively rare disease, no prospective randomized control trials have yet been performed to evaluate the efficacy and safety of each treatment modality. The clinical phenotype is heterogenous, and the management approach should be optimized according to the severity and the extent of organ involvement. Currently, there is still no international consensus or guideline on the use of immunomodulant in the treatment of DRESS/DIHS. Recently, there is a guideline for the diagnosis, management, treatment, and prevention of DRESS syndrome conducted by Spanish specialists and experts (68). The Spanish guideline made a comprehensive summary of current management considerations and provided a consensus-based stepwise management algorithm (68).

In general, the gold standard for treatment is causative drug identification and withdrawal with supportive care. Closely monitoring and assessing clinical symptoms, laboratory data, and imaging results are crucial. Multidisciplinary team care and timely consultation with other specialists are also important, especially while severe organ involvement. Systemic corticosteroids usually remain the first line of treatment, though the efficacy and risk profile of this treatment is difficult to be investigated in randomized controlled trials (68). A moderate-to-high dose (0.5–1 mg/kg/day of prednisolone equivalent dose) may be used to achieve improvement of the clinical symptoms and laboratory parameters (68, 172, 173). If the patients presented with more extensive organ involvement, a higher dose of corticosteroids may be applied. Initiation of systemic corticosteroids is recommended as first line therapy in patients with severe organ injury, such as nephritis (174), hepatitis (175), and pneumonitis if there are no contraindications (68). For patients present with more extensive organ involvement, initiating a higher dose of corticosteroids may be warranted (68). However, some disadvantages of systemic corticosteroids were reported including disease flare-ups during tapering (172), increased risk of opportunistic infection (162), and viral reactivations of HHV-6 and CMV (134). Therefore, the benefits and harms should be balanced while applying systemic corticosteroids for patients with DRESS/DIHS. For patients with a mild form of DRESS/DIHS without severe organ involvement, some authors advocate for using topical high-potency corticosteroids alone because of a lower complication rate compared with patients using systemic corticosteroids (162, 176, 177).

Other therapeutic agents have also been reported, though the evidence is mainly from small case series or case reports (see Table 3; Supplementary Table S1). Cyclosporin is another frequently reported potential therapeutic agent. Successful responses in treating patients with DRESS/DIHS have been observed in some case reports (178–181). A recent case–control study involving 26 patients (5 using cyclosporin and 21 using systemic corticosteroids) with DRESS/DIHS demonstrated cyclosporin is an effective treatment with good tolerability in patients contraindicated with systemic corticosteroids usage (183). Another study conducted by our team previously involving 8 DRESS/DIHS patients showed cyclosporin may be an effective and safe alternative treatment as a steroid-sparing agent for recalcitrant corticosteroid-dependent DRESS (182). Therefore, as per the Spanish guideline (68), cyclosporin can be considered as a second-line therapy for patients with poor control with corticosteroids or as a first-line therapy if the systemic corticosteroids are contraindicated. The recommended doses of cyclosporin use are still undetermined, but 3–5 mg/kg/day for a short course (7-day) with subsequent tapering may be considered according to current evidence. An open-labeled randomized clinical trial to compare the efficacy between high-dose cyclosporin (5 mg/kg/day) and pulse systemic corticosteroids in DRESS/DIHS is recently under enrolling (NCT04988256). More evidence of the efficacy and safety of cyclosporin may be established in the future.

**Table 3:** Summary of current alternative therapeutic options and evidence other than systemic corticosteroids.

Treatment	Mechanism	Clinical indication	Reported dose^¶^	Evidence (study design)
Topical corticosteroids alone	Inhibitory effects on a broad range of immune responses	Non-severe DRESS (absence of life-threatening organ involvement)	Potent or very potent TCS (betamethasone or clobetasol) 1–2 times/day	Case series (162, 176, 177)
Cyclosporin	Calcineurin inhibitor: inhibition of production and release of IL-2 and downstream activation of resting T-lymphocytes.	First-line therapy in early DRESS or patients contraindicated to corticosteroid Second-line therapy in corticosteroid-refractory or recurrent relapsing DRESS	3–5 mg/kg/day for 3–7 days (first-line therapy) or longerA lower dose (1–3 mg/kg/day) has also been reported	Case reports (178–180)Case series (181, 182)Retrospective case–control study (183)
IVIG	Replacement therapy for harmful autoantibodies Provides passive immunity by increasing the antibody titer with antigen–antibody reaction potential	Monotherapy in patients contraindicated to corticosteroids Add-on therapy as salvage therapy or steroid-sparing agent	0.2–2 g/kg/day for 2–5 days or monthly for 8 months as a steroid-sparing agent	Case reports (184–188)Case series (one prospective study) (189–191)
Cyclophosphamide	Alkylating agent: prevents cell division by cross-linking DNA strands and decreasing DNA synthesis	DRESS with severe internal organ (renal) involvement	750 mg/m^2^ once and relayed by oral cyclophosphamide (100 mg/day) for 6 months	Case reports (192, 193)
Plasmapheresis	Rapid removal of disease-causing autoantibodies or cells	Recurrent, relapsing, or corticosteroid-refractory DRESS with life-threatening organ involvement	4 sessions	Case reports (194–196)
Mycophenolate mofetil	IMPDH inhibitor which inhibits *de novo* guanosine nucleotide synthesis and blocks DNA synthesis	Corticosteroid-refractory DRESS with severe myocarditis (one fatal outcome)	Not specified in studies	Case reports (194, 197)
Mepolizumab	Anti-IL-5 monoclonal antibody	Recurrent, relapsing, or corticosteroid-refractory DRESS	100–300 mg monthly with single or multiple doses	Case reports (198–202)
Benralizumab	Anti-IL-5 receptor monoclonal antibody	Recurrent, relapsing, or corticosteroid-refractory DRESS	30 mg once or monthly	Case reports (198, 202–205)
Reslizumab	Anti-IL-5 monoclonal antibody	For continued use of the culprit drug	100 mg once followed by 200 mg once	Case report (only one case) (206)
Tofacitinib	Pan-JAK inhibitor	Recurrent, relapsing, life-threatening, or corticosteroid-refractory DRESS	5–10 mg/day for more than 1–10 months^§^	Case reports (207–209)

¶ Summary of doses that had been reported in the literature.

§ Higher dose (10 mg twice daily) was also reported in one case. Relapse of the symptoms was also reported in one patient taking tofacitinib for more than 10 months.

DRESS, drug reaction with eosinophilia and systemic symptoms; IL, interleukin; IMPDH, inosine monophosphate dehydrogenase; IVIG, intravenous immunoglobulin; JAK, Janus kinase; TCS, topical corticosteroids.

For intravenous immunoglobulin therapy (IVIG), inconsistent treatment results were reported in different studies (184–190). Successfully treated cases were reported from case reports and case series with variable doses and duration (0.2 to 2 g/kg/day for 2 to 5 days) as monotherapy, salvage therapy, or combined therapy with systemic corticosteroids. However, in a relatively large prospective study involving six patients, the author did not support the IVIGs as monotherapy for DRESS/DIHS because of severe adverse events and the absence of beneficial effects (191). Mycophenolate mofetil, cyclophosphamide, rituximab, and plasmapheresis are other potential modalities for management, but the evidence of their effect is primarily from case reports (192–197). These agents may be considered when the patients are refractory to the above therapies. Antiviral agents like ganciclovir and valganciclovir can be considered add-on therapies if viral reactivations with suspected of contributing to severe complications (e.g., encephalitis, hemophagocytosis, or severe erosive colitis) (68).

Target-specific biologic agents have emerged and shown promise in treating a variety of autoimmune and inflammatory diseases. Since IL-5 is one of the key pathogenic cytokines in DRESS/DIHS, IL-5/IL-5 receptor (IL-5R) blockade has been reported to be a potential strategy in the management of recalcitrant DRESS/DIHS (198). There are currently three available humanized monoclonal antibodies for IL-5/IL-5R blockade. Mepolizumab and reslizumab, directly target the IL-5 and inhibit IL-5 signaling; while benralizumab targets the α subunit of the IL-5R. Until now, 15 cases had been reported. Mepolizumab was used in seven cases (198–202), benralizumab in eight cases (198, 202–205), and reslizumab in one case of DRESS/DIHS (206). In cases using mepolizumab or reslizumab, multiple injections for 3 months may be needed to reach complete remission because relapses are common after administration (198–201, 206). Only one case demonstrated obvious clinical improvement after single dose of mepolizumab (202). By contrast, in cases using benralizumab, one subcutaneous injection at a dose of 30 mg may be sufficient in most patients (198, 203–205). However, the effectiveness of these agents for DRESS/DIHS was established based on a small number of cases.

Another signaling pathway-targeted therapy also provides a promising future for treating patients with DRESS/DIHS. By using high-output single-cell transcriptomic analysis, the Janus kinase-signal transducer and activator of transcription (JAK–STAT) signaling pathway showed significantly upregulated in a patient with DRESS/DIHS refractory to high dose corticosteroid, mycophenolate mofetil, and cyclosporine (207). A pan-JAK inhibitor with tofacitinib (up to 10 mg per day) was then administered according to the identified JAK3 and STAT1 signatures, and the disease was getting controlled well after tofacitinib treatment (207). The JAK–STAT pathway is responsible for the upstream signal for many cytokines including IL-4 (JAK1, JAK23, TYK2), IL-5 (JAK1, JAK2), and IL-13 (JAK1, TYK2), which were involved in the pathogenesis of DRESS/DIHS (210). There were another two case reports demonstrating the effectiveness of tofacitinib in treating life-threatening DRESS/DIHS complicated with myocarditis (208, 209). Although disease relapse was noticed when tofacitinib was suddenly stopped in these cases, symptoms would improve rapidly after tofacitinib was reintroduced. Long-term use and slow tapering of tofacitinib may be needed.

Moreover, OX40-OX40L and TARC/CCL17-CCR4 interaction pathways may also be potential targets for treating DRESS/DIHS. The therapeutic application of the available biologics of anti-OX40 and anti-OX40L antibodies, such as rocatinlimab and amlitelimab, has been under investigation for many immune diseases and cancers as well as Th2 dominant atopic dermatitis (87, 211). Mogamulizumab, a humanized anti-CCR4 monoclonal antibody targeting TARC/CCL17-CCR4 axis, was also applied for treating refractory or relapsed adult T cell leukemia/lymphoma and cutaneous T cell lymphoma (212). The potential therapeutic effects of these novel drugs on drug hypersensitivity reactions still need further investigation.

## 5. Conclusion

DRESS/DIHS is a rare but severe adverse drug reaction with distinct clinical features and complicated pathomechanisms. Genetic polymorphism, specific signaling pathways in T cell activation, cytokines and chemokines production, and HHV reactivation are involved in the pathogenesis of DRESS/DIHS. Some scoring systems and potential biomarkers are proposed, but not widely applied in clinical practice yet. Systemic corticosteroids are still the first line of DRESS/DIHS management, and other steroid-sparing immunomodulators may be promising treatment modalities, especially for refractory cases and those contraindicated to corticosteroids. More research is required to clarify the pathogenesis and determine the advantages and risks of the newly developed treatment modalities.

## Author contributions

C-BC, W-KH, and C-CL: conceptualization. C-BC and W-KH: methodology and investigation. C-BC and W-HC: resources, supervision, and project administration. C-BC, W-KH, C-CL, and C-WW: writing—original draft preparation. C-BC, W-KH, C-WW, C-CL, S-IH, and W-HC: writing—review and editing. C-BC, C-CL, and S-IH: visualization. All authors contributed to the article and approved the submitted version.

## Funding

This work was supported by grants from the Ministry of Science and Technology, Taiwan (MOST 108-2314-B-182A-104-MY3, 109-2320-B-182A-008-MY3, 110-2320-B-182A-014-MY3, 111-2326-B-182A-003-MY3, 111-2314-B-182A-113-MY3, and NSTC 111-2314-B-182A-113-MY3) and Chang Gung Memorial Hospital (CORPG3L0471, CORPG3L0472, and CORPG3M0361).

## Conflict of interest

The authors declare that the research was conducted in the absence of any commercial or financial relationships that could be construed as a potential conflict of interest.

## Publisher’s note

All claims expressed in this article are solely those of the authors and do not necessarily represent those of their affiliated organizations, or those of the publisher, the editors and the reviewers. Any product that may be evaluated in this article, or claim that may be made by its manufacturer, is not guaranteed or endorsed by the publisher.

## References

[1] Hausmann O Schnyder B Pichler WJ. Drug hypersensitivity reactions involving skin. Handb Exp Pharmacol. (2010) 196:29–55. doi: 10.1007/978-3-642-00663-0_2 20020258

[2] Pichler WJ. Immune pathomechanism and classification of drug hypersensitivity. Allergy. (2019) 74:1457–71. doi: 10.1111/all.13765 30843233

[3] Hama N Abe R Gibson A Phillips EJ. Drug-induced hypersensitivity syndrome (DIHS)/drug reaction with eosinophilia and systemic symptoms (DRESS): clinical features and pathogenesis. J Allergy Clin Immunol Pract. (2022) 10:1155–1167 e5. doi: 10.1016/j.jaip.2022.02.004, PMID: 35176506 PMC9201940

[4] Hung WK Chung WH. Drug reaction with eosinophilia and systemic symptoms. N Engl J Med. (2022) 387:167. doi: 10.1056/NEJMicm2116076 35830643

[5] Haruda F. Phenytoin hypersensitivity: 38 cases. Neurology. (1979) 29:1480–5. doi: 10.1212/WNL.29.11.1480, PMID: 574201

[6] Tomecki KJ Catalano CJ. Dapsone hypersensitivity. The sulfone syndrome revisited. Arch Dermatol. (1981) 117:38–9. doi: 10.1001/archderm.1981.01650010044023 6450569

[7] Singer JZ Wallace SL. The allopurinol hypersensitivity syndrome. Unnecessary morbidity and mortality. Arthritis Rheum. (1986) 29:82–7. doi: 10.1002/art.1780290111, PMID: 3947418

[8] Bocquet H Bagot M Roujeau JC. Drug-induced pseudolymphoma and drug hypersensitivity syndrome (drug rash with eosinophilia and systemic symptoms: DRESS). Semin Cutan Med Surg. (1996) 15:250–7. doi: 10.1016/S1085-5629(96)80038-1, PMID: 9069593

[9] Shiohara T Inaoka M Kano Y. Drug-induced hypersensitivity syndrome (DIHS): a reaction induced by a complex interplay among herpesviruses and antiviral and antidrug immune responses. Allergol Int. (2006) 55:1–8. doi: 10.2332/allergolint.55.1, PMID: 17075280

[10] Shiohara T Mizukawa Y. Drug-induced hypersensitivity syndrome (DiHS)/drug reaction with eosinophilia and systemic symptoms (DRESS): an update in 2019. Allergol Int. (2019) 68:301–8. doi: 10.1016/j.alit.2019.03.006, PMID: 31000444

[11] Kardaun SH Sekula P Valeyrie-Allanore L Liss Y Chu CY Creamer D. Drug reaction with eosinophilia and systemic symptoms (DRESS): an original multisystem adverse drug reaction. Results from the prospective RegiSCAR study. Br J Dermatol. (2013) 169:1071–80. doi: 10.1111/bjd.12501, PMID: 23855313

[12] Pichler WJ. Delayed drug hypersensitivity reactions. Ann Intern Med. (2003) 139:683–93. doi: 10.7326/0003-4819-139-8-200310210-00012, PMID: 14568857

[13] Rudolph MG Stanfield RL Wilson IA. How TCRs bind MHCs, peptides, and coreceptors. Annu Rev Immunol. (2006) 24:419–66. doi: 10.1146/annurev.immunol.23.021704.115658, PMID: 16551255

[14] Horton R Wilming L Rand V Lovering RC Bruford EA Khodiyar VK. Gene map of the extended human MHC. Nat Rev Genet. (2004) 5:889–99. doi: 10.1038/nrg1489 15573121

[15] Chung WH Hung SI Chen YT. Human leukocyte antigens and drug hypersensitivity. Curr Opin Allergy Clin Immunol. (2007) 7:317–23. doi: 10.1097/ACI.0b013e3282370c5f 17620823

[16] Sukasem C Sririttha S Chaichan C Nakkrut T Satapornpong P Jaruthamsophon K. Spectrum of cutaneous adverse reactions to aromatic antiepileptic drugs and human leukocyte antigen genotypes in Thai patients and meta-analysis. Pharmacogenomics J. (2021) 21:682–90. doi: 10.1038/s41397-021-00247-3, PMID: 34175889 PMC8602035

[17] McCormack M Alfirevic A Bourgeois S Farrell JJ Kasperavičiūtė D Carrington M. HLA-A*3101 and carbamazepine-induced hypersensitivity reactions in Europeans. N Engl J Med. (2011) 364:1134–43. doi: 10.1056/NEJMoa1013297, PMID: 21428769 PMC3113609

[18] Ozeki T Mushiroda T Yowang A Takahashi A Kubo M Shirakata Y. Genome-wide association study identifies HLA-A*3101 allele as a genetic risk factor for carbamazepine-induced cutaneous adverse drug reactions in Japanese population. Hum Mol Genet. (2011) 20:1034–41. doi: 10.1093/hmg/ddq537, PMID: 21149285

[19] Kim SH Lee KW Song WJ Kim SH Jee YK Lee SM. Carbamazepine-induced severe cutaneous adverse reactions and HLA genotypes in Koreans. Epilepsy Res. (2011) 97:190–7. doi: 10.1016/j.eplepsyres.2011.08.010, PMID: 21917426

[20] Genin E Chen DP Hung SI Sekula P Schumacher M Chang PY. HLA-A*31:01 and different types of carbamazepine-induced severe cutaneous adverse reactions: an international study and meta-analysis. Pharmacogenomics J. (2014) 14:281–8. doi: 10.1038/tpj.2013.40, PMID: 24322785

[21] Hung SI Chung WH Jee SH Chen WC Chang YT Lee WR. Genetic susceptibility to carbamazepine-induced cutaneous adverse drug reactions [comparative study research support, non-U.S. Gov't]. Pharmacogenet Genomics. (2006) 16:297–306. doi: 10.1097/01.fpc.0000199500.46842.4a, PMID: 16538176

[22] Mockenhaupt M Wang CW Hung SI Sekula P Schmidt AH Pan RY. HLA-B*57:01 confers genetic susceptibility to carbamazepine-induced SJS/TEN in Europeans. Allergy. (2019) 74:2227–30. doi: 10.1111/all.13821, PMID: 30972788

[23] Ramírez E Bellón T Tong HY Borobia AM de Abajo FJ Lerma V. Significant HLA class I type associations with aromatic antiepileptic drug (AED)-induced SJS/TEN are different from those found for the same AED-induced DRESS in the Spanish population. Pharmacol Res. (2017) 115:168–78. doi: 10.1016/j.phrs.2016.11.027, PMID: 27888155

[24] Mortazavi H Rostami A Firooz A Esmaili N Ghiasi M Lajevardi V. Association between human leukocyte antigens and cutaneous adverse drug reactions to antiepileptics and antibiotics in the Iranian population. Dermatol Ther. (2022) 35:e15393. doi: 10.1111/dth.15393, PMID: 35187767

[25] Wong CSM Yap DYH Ip P Wong WHS Chua GT Yeung CK. HLA-B*15:11 status and carbamazepine-induced severe cutaneous adverse drug reactions in HLA-B*15:02 negative Chinese. Int J Dermatol. (2022) 61:184–90. doi: 10.1111/ijd.15792, PMID: 34553372

[26] Yampayon K Sukasem C Limwongse C Chinvarun Y Tempark T Rerkpattanapipat T. Influence of genetic and non-genetic factors on phenytoin-induced severe cutaneous adverse drug reactions. Eur J Clin Pharmacol. (2017) 73:855–65. doi: 10.1007/s00228-017-2250-2, PMID: 28391407

[27] Tassaneeyakul W Prabmeechai N Sukasem C Kongpan T Konyoung P Chumworathayi P. Associations between HLA class I and cytochrome P450 2C9 genetic polymorphisms and phenytoin-related severe cutaneous adverse reactions in a Thai population. Pharmacogenet Genomics. (2016) 26:225–34. doi: 10.1097/FPC.0000000000000211, PMID: 26928377

[28] Manuyakorn W Likkasittipan P Wattanapokayakit S Suvichapanich S Inunchot W Wichukchinda N. Association of HLA genotypes with phenytoin induced severe cutaneous adverse drug reactions in Thai children. Epilepsy Res. (2020) 162:106321. doi: 10.1016/j.eplepsyres.2020.106321, PMID: 32272329

[29] Chang CC Ng CC Too CL Choon SE Lee CK Chung WH. Association of HLA-B*15:13 and HLA-B*15:02 with phenytoin-induced severe cutaneous adverse reactions in a Malay population. Pharmacogenomics J. (2017) 17:170–3. doi: 10.1038/tpj.2016.10, PMID: 26927288

[30] Chung WH Chang WC Lee YS Wu YY Yang CH Ho HC. Genetic variants associated with phenytoin-related severe cutaneous adverse reactions. JAMA. (2014) 312:525–34. doi: 10.1001/jama.2014.7859, PMID: 25096692

[31] Kim BK Jung JW Kim TB Chang YS Park HS Moon J. HLA-A*31:01 and lamotrigine-induced severe cutaneous adverse drug reactions in a Korean population. Ann Allergy Asthma Immunol. (2017) 118:629–30. doi: 10.1016/j.anai.2017.02.011 28351624

[32] Kazeem GR Cox C Aponte J Messenheimer J Brazell C Nelsen AC. High-resolution HLA genotyping and severe cutaneous adverse reactions in lamotrigine-treated patients. Pharmacogenet Genomics. (2009) 19:661–5. doi: 10.1097/FPC.0b013e32832c347d, PMID: 19668019

[33] Hung SI Chung WH Liou LB Chu CC Lin M Huang HP. HLA-B*5801 allele as a genetic marker for severe cutaneous adverse reactions caused by allopurinol. Proc Natl Acad Sci U S A. (2005) 102:4134–9. doi: 10.1073/pnas.0409500102, PMID: 15743917 PMC554812

[34] Tassaneeyakul W Jantararoungtong T Chen P Lin PY Tiamkao S Khunarkornsiri U. Strong association between HLA-B*5801 and allopurinol-induced Stevens-Johnson syndrome and toxic epidermal necrolysis in a Thai population. Pharmacogenet Genomics. (2009) 19:704–9. doi: 10.1097/FPC.0b013e328330a3b8, PMID: 19696695

[35] Kaniwa N Saito Y Aihara M Matsunaga K Tohkin M Kurose K. HLA-B locus in Japanese patients with anti-epileptics and allopurinol-related Stevens-Johnson syndrome and toxic epidermal necrolysis. Pharmacogenomics. (2008) 9:1617–22. doi: 10.2217/14622416.9.11.1617, PMID: 19018717

[36] Kang HR Jee YK Kim YS Lee CH Jung JW Kim SH. Positive and negative associations of HLA class I alleles with allopurinol-induced SCARs in Koreans. Pharmacogenet Genomics. (2011) 21:303–7. doi: 10.1097/FPC.0b013e32834282b8, PMID: 21301380

[37] Kim MY Yun J Kang DY Kim TH Oh MK Lee S. HLA-A*24:02 increase the risk of allopurinol-induced drug reaction with eosinophilia and systemic symptoms in HLA-B*58:01 carriers in a Korean population; a multicenter cross-sectional case-control study. Clin Transl Allergy. (2022) 12:e12193. doi: 10.1002/clt2.12193, PMID: 36176736 PMC9478421

[38] Saag M Balu R Phillips E Brachman P Martorell C Burman W. High sensitivity of human leukocyte antigen-b*5701 as a marker for immunologically confirmed abacavir hypersensitivity in white and black patients. Clin Infect Dis. (2008) 46:1111–8. doi: 10.1086/529382, PMID: 18444831

[39] Hetherington S Hughes AR Mosteller M Shortino D Baker KL Spreen W. Genetic variations in HLA-B region and hypersensitivity reactions to abacavir. Lancet. (2002) 359:1121–2. doi: 10.1016/S0140-6736(02)08158-8, PMID: 11943262

[40] Mallal S Nolan D Witt C Masel G Martin AM Moore C. Association between presence of HLA-B*5701, HLA-DR7, and HLA-DQ3 and hypersensitivity to HIV-1 reverse-transcriptase inhibitor abacavir. Lancet. (2002) 359:727–32. doi: 10.1016/S0140-6736(02)07873-X, PMID: 11888582

[41] Hughes AR Mosteller M Bansal AT Davies K Haneline SA Lai EH. Association of genetic variations in HLA-B region with hypersensitivity to abacavir in some, but not all, populations. Pharmacogenomics. (2004) 5:203–11. doi: 10.1517/phgs.5.2.203.27481 15016610

[42] Manglani MV Gabhale YR Lala MM Sekhar R More D. HLA-B*5701 allele in HIV-infected Indian children and its association with Abacavir hypersensitivity. Indian Pediatr. (2018) 55:140–1. doi: 10.1007/s13312-018-1248-x, PMID: 29242412

[43] Martin AM Nolan D James I Cameron P Keller J Moore C. Predisposition to nevirapine hypersensitivity associated with HLA-DRB1*0101 and abrogated by low CD4 T-cell counts. AIDS. (2005) 19:97–9. doi: 10.1097/00002030-200501030-00014, PMID: 15627041

[44] Chantarangsu S Mushiroda T Mahasirimongkol S Kiertiburanakul S Sungkanuparph S Manosuthi W. HLA-B*3505 allele is a strong predictor for nevirapine-induced skin adverse drug reactions in HIV-infected Thai patients. Pharmacogenet Genomics. (2009) 19:139–46. doi: 10.1097/FPC.0b013e32831d0faf, PMID: 19104471

[45] Littera R Carcassi C Masala A Piano P Serra P Ortu F. HLA-dependent hypersensitivity to nevirapine in Sardinian HIV patients. AIDS. (2006) 20:1621–6. doi: 10.1097/01.aids.0000238408.82947.09, PMID: 16868443

[46] Gatanaga H Yazaki H Tanuma J Honda M Genka I Teruya K. HLA-Cw8 primarily associated with hypersensitivity to nevirapine. AIDS. (2007) 21:264–5. doi: 10.1097/QAD.0b013e32801199d9, PMID: 17197830

[47] Carr DF Chaponda M Jorgensen AL Castro EC van Oosterhout JJ Khoo SH. Association of human leukocyte antigen alleles and nevirapine hypersensitivity in a Malawian HIV-infected population. Clin Infect Dis. (2013) 56:1330–9. doi: 10.1093/cid/cit021, PMID: 23362284 PMC3616517

[48] Gao S Gui XE Liang K Liu Z Hu J Dong B. HLA-dependent hypersensitivity reaction to nevirapine in Chinese Han HIV-infected patients. AIDS Res Hum Retrovir. (2012) 28:540–3. doi: 10.1089/aid.2011.0107, PMID: 21902584

[49] Lefebvre M Walencik A Allavena C Billaud E Kassi A Cesbron A. Rate of DRESS syndrome with Raltegravir and role of the HLA-B*53: 01 allele. J Acquir Immune Defic Syndr. (2020) 85:e77–80. doi: 10.1097/QAI.0000000000002474, PMID: 33136758

[50] Thomas M Hopkins C Duffy E Lee D Loulergue P Ripamonti D. Association of the HLA-B*53:01 allele with drug reaction with eosinophilia and systemic symptoms (DRESS) syndrome during treatment of HIV infection with Raltegravir. Clin Infect Dis. (2017) 64:1198–203. doi: 10.1093/cid/cix096, PMID: 28369189 PMC5399947

[51] Zhang FR Liu H Irwanto A Fu XA Li Y Yu GQ. HLA-B*13:01 and the dapsone hypersensitivity syndrome. N Engl J Med. (2013) 369:1620–8. doi: 10.1056/NEJMoa1213096, PMID: 24152261

[52] Krismawati H Irwanto A Pongtiku A Irwan ID Maladan Y Sitanggang YA. Validation study of HLA-B*13:01 as a biomarker of dapsone hypersensitivity syndrome in leprosy patients in Indonesia. PLoS Negl Trop Dis. (2020) 14:e0008746. doi: 10.1371/journal.pntd.0008746, PMID: 33064728 PMC7592909

[53] Park HJ Park JW Kim SH Choi SY Kim HK Jung CG. The HLA-B*13:01 and the dapsone hypersensitivity syndrome in Korean and Asian populations: genotype- and meta-analyses. Expert Opin Drug Saf. (2020) 19:1349–56. doi: 10.1080/14740338.2020.1796965, PMID: 32700588

[54] Satapornpong P Pratoomwun J Rerknimitr P Klaewsongkram J Nakkam N Rungrotmongkol T. HLA-B*13:01 is a predictive marker of Dapsone-induced severe cutaneous adverse reactions in Thai patients. Front Immunol. (2021) 12:661135. doi: 10.3389/fimmu.2021.661135, PMID: 34017337 PMC8130671

[55] Chen WT Wang CW Lu CW Chen CB Lee HE Hung SI. The function of HLA-B*13:01 involved in the Pathomechanism of Dapsone-induced severe cutaneous adverse reactions. J Invest Dermatol. (2018) 138:1546–54. doi: 10.1016/j.jid.2018.02.004, PMID: 29458119

[56] Wang CW Tassaneeyakul W Chen CB Chen WT Teng YC Huang CY. Taiwan/Asian severe cutaneous adverse reaction C. whole genome sequencing identifies genetic variants associated with co-trimoxazole hypersensitivity in Asians. J Allergy Clin Immunol. (2021) 147:1402–12. doi: 10.1016/j.jaci.2020.08.003, PMID: 32791162

[57] Sukasem C Pratoomwun J Satapornpong P Klaewsongkram J Rerkpattanapipat T Rerknimitr P. Genetic Association of co-Trimoxazole-Induced Severe Cutaneous Adverse Reactions is Phenotype-Specific: HLA class I genotypes and haplotypes. Clin Pharmacol Ther. (2020) 108:1078–89. doi: 10.1002/cpt.1915, PMID: 32452529

[58] Nakkam N Saksit N Konyoung P Amornpinyo W Khunarkornsiri U Purimart D. Associations of HLA and drug-metabolizing enzyme genes in co-trimoxazole-induced severe cutaneous adverse reactions. Drug Metab Pharmacokinet. (2022) 47:100480. doi: 10.1016/j.dmpk.2022.100480, PMID: 36379177

[59] Yang F Gu B Zhang L Xuan J Luo H Zhou P. HLA-B*13:01 is associated with salazosulfapyridine-induced drug rash with eosinophilia and systemic symptoms in Chinese Han population. Pharmacogenomics. (2014) 15:1461–9. doi: 10.2217/pgs.14.69, PMID: 25303297

[60] Nakamura R Ozeki T Hirayama N Sekine A Yamashita T Mashimo Y. Association of HLA-A*11:01 with sulfonamide-related severe cutaneous adverse reactions in Japanese patients. J Invest Dermatol. (2020) 140:1659–1662.e6. doi: 10.1016/j.jid.2019.12.025, PMID: 31981579

[61] Rutkowski K Taylor C Wagner A. HLA B62 as a possible risk factor for drug reaction with eosinophilia and systemic symptoms to piperacillin/tazobactam. J Allergy Clin Immunol Pract. (2017) 5:829–30. doi: 10.1016/j.jaip.2016.10.008, PMID: 27914818

[62] Konvinse KC Trubiano JA Pavlos R James I Shaffer CM Bejan CA. HLA-A*32:01 is strongly associated with vancomycin-induced drug reaction with eosinophilia and systemic symptoms. J Allergy Clin Immunol. (2019) 144:183–92. doi: 10.1016/j.jaci.2019.01.045, PMID: 30776417 PMC6612297

[63] Bellón T Lerma V Guijarro J Ramírez E Martínez C Escudero C. LTT and HLA testing as diagnostic tools in Spanish vancomycin-induced DRESS cases: A case-control study. Front Pharmacol. (2022) 13:959321. doi: 10.3389/fphar.2022.959321, PMID: 36339612 PMC9631441

[64] Wang CW Lin WC Chen WT Chen CB Lu CW Hou HH. Associations of HLA-A and HLA-B with vancomycin-induced drug reaction with eosinophilia and systemic symptoms in the Han-Chinese population. Front Pharmacol. (2022) 13:954596. doi: 10.3389/fphar.2022.954596 36506572 PMC9732226

[65] Balas A Ramírez E Trigo E Cabañas R Fiandor A Arsuaga M. HLA-A∗68, −A∗11:01, and -A∗29:02 alleles are strongly associated with benznidazole-induced maculopapular exanthema (MPE)/DRESS. J Allergy Clin Immunol Pract. (2020) 8:3198–3200.e3. doi: 10.1016/j.jaip.2020.05.004, PMID: 32417447

[66] Salehi T Fleet AP Hissaria P Carroll RP Au PC. Human leukocyte antigen association with azathioprine-induced drug hypersensitivity reactions in patients with anti-neutrophil cytoplasmic antibody associated vasculitis. Hum Immunol. (2023) 84:196–8. doi: 10.1016/j.humimm.2022.12.006, PMID: 36610806

[67] Saper VE Ombrello MJ Tremoulet AH Montero-Martin G Prahalad S Canna S. Severe delayed hypersensitivity reactions to IL-1 and IL-6 inhibitors link to common HLA-DRB1*15 alleles. Ann Rheum Dis. (2022) 81:406–15. doi: 10.1136/annrheumdis-2021-220578, PMID: 34789453 PMC10564446

[68] Cabanas R Ramirez E Sendagorta E Alamar R Barranco R Blanca-Lopez N. Spanish guidelines for diagnosis, management, treatment, and prevention of DRESS syndrome. J Investig Allergol Clin Immunol. (2020) 30:229–53. doi: 10.18176/jiaci.0480, PMID: 31932268

[69] Ko TM Tsai CY Chen SY Chen KS Yu KH Chu CS. Use of HLA-B*58:01 genotyping to prevent allopurinol induced severe cutaneous adverse reactions in Taiwan: national prospective cohort study. BMJ. (2015) 351:h4848. doi: 10.1136/bmj.h4848, PMID: 26399967 PMC4579807

[70] Hemmer B Kondo T Gran B Pinilla C Cortese I Pascal J. Minimal peptide length requirements for CD4(+) T cell clones--implications for molecular mimicry and T cell survival. Int Immunol. (2000) 12:375–83. doi: 10.1093/intimm/12.3.375, PMID: 10700472

[71] Pavlos R Mallal S Ostrov D Buus S Metushi I Peters B. T cell-mediated hypersensitivity reactions to drugs. Annu Rev Med. (2015) 66:439–54. doi: 10.1146/annurev-med-050913-022745, PMID: 25386935 PMC4295772

[72] Padovan E Mauri-Hellweg D Pichler WJ Weltzien HU. T cell recognition of penicillin G: structural features determining antigenic specificity. Eur J Immunol. (1996) 26:42–8. doi: 10.1002/eji.1830260107, PMID: 8566082

[73] Pichler WJ. Pharmacological interaction of drugs with antigen-specific immune receptors: the p-i concept. Curr Opin Allergy Clin Immunol. (2002) 2:301–5. doi: 10.1097/00130832-200208000-00003, PMID: 12130944

[74] Schnyder B Mauri-Hellweg D Zanni M Bettens F Pichler WJ. Direct, MHC-dependent presentation of the drug sulfamethoxazole to human alphabeta T cell clones. J Clin Invest. (1997) 100:136–41. doi: 10.1172/JCI119505, PMID: 9202065 PMC508173

[75] Zanni MP von Greyerz S Schnyder B Brander KA Frutig K Hari Y. HLA-restricted, processing- and metabolism-independent pathway of drug recognition by human alpha beta T lymphocytes. J Clin Invest. (1998) 102:1591–8. doi: 10.1172/JCI3544, PMID: 9788973 PMC509010

[76] Zanni MP von Greyerz S Schnyder B Wendland T Pichler WJ. Allele-unrestricted presentation of lidocaine by HLA-DR molecules to specific alphabeta+ T cell clones. Int Immunol. (1998) 10:507–15. doi: 10.1093/intimm/10.4.507, PMID: 9620607

[77] Wei CY Chung WH Huang HW Chen YT Hung SI. Direct interaction between HLA-B and carbamazepine activates T cells in patients with Stevens-Johnson syndrome. J Allergy Clin Immunol. (2012) 129:1562–9.e5. doi: 10.1016/j.jaci.2011.12.990, PMID: 22322005

[78] Jiang H Wang CW Wang Z Dai Y Zhu Y Lee YS. Functional and structural characteristics of HLA-B*13:01-mediated specific T cells reaction in dapsone-induced drug hypersensitivity. J Biomed Sci. (2022) 29:58. The authors declare that they have no competing interests. doi: 10.1186/s12929-022-00845-8 35964029 PMC9375929

[79] Ostrov DA Grant BJ Pompeu YA Sidney J Harndahl M Southwood S. Drug hypersensitivity caused by alteration of the MHC-presented self-peptide repertoire. Proc Natl Acad Sci. (2012) 109:9959–64. doi: 10.1073/pnas.1207934109, PMID: 22645359 PMC3382472

[80] Illing PT Vivian JP Dudek NL Kostenko L Chen Z Bharadwaj M. Immune self-reactivity triggered by drug-modified HLA-peptide repertoire [research support, non-U.S. Gov't]. Nature. (2012) 486:554–8. doi: 10.1038/nature11147, PMID: 22722860

[81] Norcross MA Luo S Lu L Boyne MT Gomarteli M Rennels AD. Abacavir induces loading of novel self-peptides into HLA-B*57: 01: an autoimmune model for HLA-associated drug hypersensitivity. AIDS. (2012) 26:F21–9. doi: 10.1097/QAD.0b013e328355fe8f, PMID: 22617051 PMC4155923

[82] Callan HE Jenkins RE Maggs JL Lavergne SN Clarke SE Naisbitt DJ. Multiple adduction reactions of nitroso sulfamethoxazole with cysteinyl residues of peptides and proteins: implications for hapten formation. Chem Res Toxicol. (2009) 22:937–48. doi: 10.1021/tx900034r, PMID: 19358516

[83] Watkins S Pichler WJ. Activating interactions of sulfanilamides with T cell receptors. Open J Immunol. (2013) 3:139–57. doi: 10.4236/oji.2013.33019, PMID: 36172594 PMC7613643

[84] Hsu YO Lu KL Fu Y Wang CW Lu CW Lin YF. The roles of Immunoregulatory networks in severe drug hypersensitivity. Front Immunol. (2021) 12:597761. doi: 10.3389/fimmu.2021.597761, PMID: 33717075 PMC7953830

[85] Ishii N Takahashi T Soroosh P Sugamura K. OX40–OX40 ligand interaction in T-cell-mediated immunity and immunopathology. Adv Immunol. (2010) 105:63–98. doi: 10.1016/S0065-2776(10)05003-0 20510730

[86] Willoughby J Griffiths J Tews I Cragg MS. OX40: structure and function—what questions remain? Mol Immunol. (2017) 83:13–22. doi: 10.1016/j.molimm.2017.01.006, PMID: 28092803

[87] Fu Y Lin Q Zhang Z Zhang L. Therapeutic strategies for the costimulatory molecule OX40 in T-cell-mediated immunity. Acta Pharm Sin B. (2020) 10:414–33. doi: 10.1016/j.apsb.2019.08.010, PMID: 32140389 PMC7049610

[88] Miyagawa F Nakamura Y Miyashita K Iioka H Himuro Y Ogawa K. Preferential expression of CD134, an HHV-6 cellular receptor, on CD4T cells in drug-induced hypersensitivity syndrome (DIHS)/drug reaction with eosinophilia and systemic symptoms (DRESS). J Dermatol Sci. (2016) 83:151–4. doi: 10.1016/j.jdermsci.2016.05.001, PMID: 27174092

[89] Miyagawa F Nakamura-Nishimura Y Kanatani Y Asada H. Correlation between expression of CD134, a human herpesvirus 6 cellular receptor, on CD4+ T cells and Th2-type immune responses in drug-induced hypersensitivity syndrome/drug reaction with eosinophilia and systemic symptoms. Acta Derm Venereol. (2020) 100:adv00102. doi: 10.2340/00015555-3465, PMID: 32201900 PMC9128879

[90] Tang H Serada S Kawabata A Ota M Hayashi E Naka T. CD134 is a cellular receptor specific for human herpesvirus-6B entry. Proc Natl Acad Sci U S A. (2013) 110:9096–9. doi: 10.1073/pnas.1305187110, PMID: 23674671 PMC3670305

[91] Mitsui Y Shinkuma S Nakamura-Nishimura Y Ommori R Ogawa K Miyagawa F. Serum soluble OX40 as a diagnostic and prognostic biomarker for drug-induced hypersensitivity syndrome/drug reaction with eosinophilia and systemic symptoms. J Allergy Clin Immunol Pract. (2022) 10:558–565 e4. doi: 10.1016/j.jaip.2021.10.042, PMID: 34757063

[92] Nakamura-Nishimura Y Miyagawa F Miyashita K Ommori R Azukizawa H Asada H. Serum thymus and activation-regulated chemokine is associated with the severity of drug reaction with eosinophilia and systemic symptoms/drug-induced hypersensitivity syndrome. Br J Dermatol. (2018) 178:1430–2. doi: 10.1111/bjd.16132, PMID: 29150836

[93] Lee ES Kiuchi Y Inomata N Sueki H. Increased expression of human herpes virus 6 receptor CD134/OX40 in skin lesions of patients with drug-induced hypersensitivity syndrome/drug reaction with eosinophilia and systemic symptoms. J Dermatol. (2022) 49:e221–3. doi: 10.1111/1346-8138.16341, PMID: 36121111

[94] Postow MA Sidlow R Hellmann MD. Immune-related adverse events associated with immune checkpoint blockade. N Engl J Med. (2018) 378:158–68. doi: 10.1056/NEJMra1703481 29320654

[95] Keir ME Butte MJ Freeman GJ Sharpe AH. PD-1 and its ligands in tolerance and immunity. Annu Rev Immunol. (2008) 26:677–704. doi: 10.1146/annurev.immunol.26.021607.090331 18173375 PMC10637733

[96] Zamani MR Aslani S Salmaninejad A Javan MR Rezaei N. PD-1/PD-L and autoimmunity: A growing relationship. Cell Immunol. (2016) 310:27–41. doi: 10.1016/j.cellimm.2016.09.009, PMID: 27660198

[97] Hosseini A Gharibi T Marofi F Babaloo Z Baradaran B. CTLA-4: from mechanism to autoimmune therapy. Int Immunopharmacol. (2020) 80:106221. doi: 10.1016/j.intimp.2020.106221 32007707

[98] Lu J Thuraisingam T Chergui M Nguyen K. Nivolumab-associated DRESS syndrome: A case report. JAAD Case Rep. (2019) 5:216–8. doi: 10.1016/j.jdcr.2018.11.017, PMID: 30809563 PMC6374958

[99] Ai L Gao J Zhao S Li Q Cui YH Liu Q. Nivolumab-associated DRESS in a genetic susceptible individual. J Immunother Cancer. (2021) 9:e002879. doi: 10.1136/jitc-2021-002879 34599025 PMC8488716

[100] Mirza S Hill E Ludlow SP Nanjappa S. Checkpoint inhibitor-associated drug reaction with eosinophilia and systemic symptom syndrome. Melanoma Res. (2017) 27:271–3. doi: 10.1097/CMR.0000000000000326, PMID: 28146044

[101] Voskens CJ Goldinger SM Loquai C Robert C Kaehler KC Berking C. The price of tumor control: an analysis of rare side effects of anti-CTLA-4 therapy in metastatic melanoma from the ipilimumab network. PLoS One. (2013) 8:e53745. doi: 10.1371/journal.pone.0053745, PMID: 23341990 PMC3544906

[102] Ford M Sahbudin I Filer A Steven N Fisher BA. High proportion of drug hypersensitivity reactions to sulfasalazine following its use in anti-PD-1-associated inflammatory arthritis. Rheumatology. (2018) 57:2244–6. doi: 10.1093/rheumatology/key234, PMID: 30107548

[103] Naisbitt DJ Olsson-Brown A Gibson A Meng X Ogese MO Tailor A. Immune dysregulation increases the incidence of delayed-type drug hypersensitivity reactions. Allergy. (2020) 75:781–97. doi: 10.1111/all.14127, PMID: 31758810

[104] Hammond S Thomson P Meng X Naisbitt D. In-vitro approaches to predict and study T-cell mediated hypersensitivity to drugs. Front Immunol. (2021) 12:630530. doi: 10.3389/fimmu.2021.630530, PMID: 33927714 PMC8076677

[105] Hammond S Olsson-Brown A Grice S Gibson A Gardner J Castrejon-Flores JL. Checkpoint inhibition reduces the threshold for drug-specific T-cell priming and increases the incidence of sulfasalazine hypersensitivity. Toxicol Sci. (2022) 186:58–69. doi: 10.1093/toxsci/kfab144, PMID: 34850240 PMC8883351

[106] Saito N Abe R Yoshioka N Murata J Fujita Y Shimizu H. Prolonged elevation of serum granulysin in drug-induced hypersensitivity syndrome. Br J Dermatol. (2012) 167:452–3. doi: 10.1111/j.1365-2133.2012.10921.x, PMID: 22384988

[107] Weinborn M Barbaud A Truchetet F Beurey P Germain L Cribier B. Histopathological study of six types of adverse cutaneous drug reactions using granulysin expression. Int J Dermatol. (2016) 55:1225–33. doi: 10.1111/ijd.13350, PMID: 27421110

[108] Yang F Chen SA Wu X Zhu Q Luo X. Overexpression of cytotoxic proteins correlates with liver function impairment in patients with drug reaction with eosinophilia and systemic symptoms (DRESS). Eur J Dermatol. (2018) 28:13–25. doi: 10.1684/ejd.2017.3211, PMID: 29521632

[109] Beeler A Engler O Gerber BO Pichler WJ. Long-lasting reactivity and high frequency of drug-specific T cells after severe systemic drug hypersensitivity reactions. J Allergy Clin Immunol. (2006) 117:455–62. doi: 10.1016/j.jaci.2005.10.030, PMID: 16461148

[110] Picard D Janela B Descamps V D'Incan M Courville P Jacquot S. Drug reaction with eosinophilia and systemic symptoms (DRESS): a multiorgan antiviral T cell response. Sci Transl Med. (2010) 2:116. doi: 10.1126/scitranslmed.3001116, PMID: 20739682

[111] Srinoulprasert Y Kumkamthornkul P Tuchinda P Wongwiangjunt S Sathornsumetee S Jongjaroenprasert K. Differential cytokine profiles produced by anti-epileptic drug re-exposure of peripheral blood mononuclear cells derived from severe anti-epileptic drug patients and non-allergic controls. Cytokine. (2022) 157:155951. doi: 10.1016/j.cyto.2022.155951, PMID: 35772364

[112] Yoshikawa T Fujita A Yagami A Suzuki K Matsunaga K Ihira M. Human herpesvirus 6 reactivation and inflammatory cytokine production in patients with drug-induced hypersensitivity syndrome. J Clin Virol. (2006) 37:S92–6. doi: 10.1016/S1386-6532(06)70019-1, PMID: 17276377

[113] Mizukawa Y Kimishima M Aoyama Y Shiohara T. Predictive biomarkers for cytomegalovirus reactivation before and after immunosuppressive therapy: A single-institution retrospective long-term analysis of patients with drug-induced hypersensitivity syndrome (DiHS)/drug reaction with eosinophilia and systemic syndrome (DRESS). Int J Infect Dis. (2020) 100:239–46. doi: 10.1016/j.ijid.2020.08.078, PMID: 32891735

[114] Tsai YG Liou JH Hung SI Chen CB Chiu TM Wang CW. Increased type 2 innate lymphoid cells in patients with drug reaction with eosinophilia and systemic symptoms syndrome. J Invest Dermatol. (2019) 139:1722–31. doi: 10.1016/j.jid.2018.10.048, PMID: 30735685

[115] Yang CW Cho YT Hsieh YC Hsu SH Chen KL Chu CY. The interferon-γ-induced protein 10/CXCR3 axis is associated with human herpesvirus-6 reactivation and the development of sequelae in drug reaction with eosinophilia and systemic symptoms. Br J Dermatol. (2020) 183:909–19. doi: 10.1111/bjd.18942, PMID: 32037509

[116] Ogawa K Morito H Hasegawa A Daikoku N Miyagawa F Okazaki A. Identification of thymus and activation-regulated chemokine (TARC/CCL17) as a potential marker for early indication of disease and prediction of disease activity in drug-induced hypersensitivity syndrome (DIHS)/drug rash with eosinophilia and systemic symptoms (DRESS). J Dermatol Sci. (2013) 69:38–43. doi: 10.1016/j.jdermsci.2012.10.002, PMID: 23141052

[117] Ogawa K Morito H Hasegawa A Miyagawa F Kobayashi N Watanabe H. Elevated serum thymus and activation-regulated chemokine (TARC/CCL17) relates to reactivation of human herpesvirus 6 in drug reaction with eosinophilia and systemic symptoms (DRESS)/drug-induced hypersensitivity syndrome (DIHS). Br J Dermatol. (2014) 171:425–7. doi: 10.1111/bjd.12948, PMID: 24601914

[118] Choquet-Kastylevsky G Intrator L Chenal C Bocquet H Revuz J Roujeau JC. Increased levels of interleukin 5 are associated with the generation of eosinophilia in drug-induced hypersensitivity syndrome. Br J Dermatol. (1998) 139:1026–32. doi: 10.1046/j.1365-2133.1998.02559.x, PMID: 9990366

[119] Teraki Y Fukuda T. Skin-homing IL-13-producing T cells expand in the circulation of patients with drug rash with eosinophilia and systemic symptoms. Dermatology. (2017) 233:242–9. doi: 10.1159/000475546, PMID: 28601883

[120] Greenfeder S Umland SP Cuss FM Chapman RW Egan RW. Th2 cytokines and asthma. The role of interleukin-5 in allergic eosinophilic disease. Respir Res. (2001) 2:71–9. doi: 10.1186/rr41, PMID: 11686868 PMC59571

[121] Yawalkar N Shrikhande M Hari Y Nievergelt H Braathen LR Pichler WJ. Evidence for a role for IL-5 and eotaxin in activating and recruiting eosinophils in drug-induced cutaneous eruptions. J Allergy Clin Immunol. (2000) 106:1171–6. doi: 10.1067/mai.2000.110922, PMID: 11112902

[122] Catherine J Roufosse F. What does elevated TARC/CCL17 expression tell us about eosinophilic disorders? Semin Immunopathol. (2021) 43:439–58. doi: 10.1007/s00281-021-00857-w, PMID: 34009399 PMC8132044

[123] Imai T Nagira M Takagi S Kakizaki M Nishimura M Wang J. Selective recruitment of CCR4-bearing Th2 cells toward antigen-presenting cells by the CC chemokines thymus and activation-regulated chemokine and macrophage-derived chemokine. Int Immunol. (1999) 11:81–8. doi: 10.1093/intimm/11.1.81, PMID: 10050676

[124] Komatsu-Fujii T Kaneko S Chinuki Y Suyama Y Ohta M Niihara H. Serum TARC levels are strongly correlated with blood eosinophil count in patients with drug eruptions. Allergol Int. (2017) 66:116–22. doi: 10.1016/j.alit.2016.06.003, PMID: 27497618

[125] Takatori H Makita S Ito T Matsuki A Nakajima H. Regulatory mechanisms of IL-33-ST2-mediated allergic inflammation. Front Immunol. (2018) 9:2004. doi: 10.3389/fimmu.2018.02004, PMID: 30233590 PMC6131616

[126] Nishio D Izu K Kabashima K Tokura Y. T cell populations propagating in the peripheral blood of patients with drug eruptions. J Dermatol Sci. (2007) 48:25–33. doi: 10.1016/j.jdermsci.2007.05.013, PMID: 17601705

[127] Chen YC Chiang HH Cho YT Chang CY Chen KL Yang CW. Human herpes virus reactivations and dynamic cytokine profiles in patients with cutaneous adverse drug reactions --a prospective comparative study. Allergy. (2015) 70:568–75. doi: 10.1111/all.12602, PMID: 25727950

[128] Chung WH Hung SI Yang JY Su SC Huang SP Wei CY. Granulysin is a key mediator for disseminated keratinocyte death in Stevens-Johnson syndrome and toxic epidermal necrolysis. Nat Med. (2008) 14:1343–50. doi: 10.1038/nm.1884, PMID: 19029983

[129] Gibson A Deshpande P Campbell CN Krantz MS Mukherjee E Mockenhaupt M. Updates on the immunopathology and genomics of severe cutaneous adverse drug reactions. J Allergy Clin Immunol. (2023) 151:289–300.e4. doi: 10.1016/j.jaci.2022.12.005, PMID: 36740326 PMC9976545

[130] Abe R Shimizu T Shibaki A Nakamura H Watanabe H Shimizu H. Toxic epidermal necrolysis and Stevens-Johnson syndrome are induced by soluble Fas ligand. Am J Pathol. (2003) 162:1515–20. doi: 10.1016/S0002-9440(10)64284-8, PMID: 12707034 PMC1851208

[131] Yang CW Cho YT Hsieh YC Hsu SH Chen KL Chu CY. The interferon-gamma-induced protein 10/CXCR3 axis is associated with human herpesvirus-6 reactivation and the development of sequelae in drug reaction with eosinophilia and systemic symptoms. Br J Dermatol. (2020) 183:909–19. doi: 10.1111/bjd.18942, PMID: 32037509

[132] Kim MR Manoukian R Yeh R Silbiger SM Danilenko DM Scully S. Transgenic overexpression of human IL-17E results in eosinophilia, B-lymphocyte hyperplasia, and altered antibody production. Blood. (2002) 100:2330–40. doi: 10.1182/blood-2002-01-0012, PMID: 12239140

[133] Abbas AK Trotta E Marson A Bluestone JA. Revisiting IL-2: biology and therapeutic prospects. Sci Immunol. (2018) 3:482. doi: 10.1126/sciimmunol.aat1482, PMID: 29980618

[134] Ishida T Kano Y Mizukawa Y Shiohara T. The dynamics of herpesvirus reactivations during and after severe drug eruptions: their relation to the clinical phenotype and therapeutic outcome. Allergy. (2014) 69:798–805. doi: 10.1111/all.12410, PMID: 24749495 PMC4112819

[135] Shiohara T Iijima M Ikezawa Z Hashimoto K. The diagnosis of a DRESS syndrome has been sufficiently established on the basis of typical clinical features and viral reactivations. Br J Dermatol. (2007) 156:1083–4. doi: 10.1111/j.1365-2133.2007.07807.x, PMID: 17381452

[136] Ushigome Y Kano Y Hirahara K Shiohara T. Human herpesvirus 6 reactivation in drug-induced hypersensitivity syndrome and DRESS validation score. Am J Med. (2012) 125:e9–e10. doi: 10.1016/j.amjmed.2011.10.027, PMID: 22727240

[137] Ang CC Wang YS Yoosuff EL Tay YK. Retrospective analysis of drug-induced hypersensitivity syndrome: a study of 27 patients. J Am Acad Dermatol. (2010) 63:219–27. doi: 10.1016/j.jaad.2009.08.050, PMID: 20605253

[138] Otani N Okuno T. Human herpesvirus 6 infection of CD4+ T-cell subsets. Microbiol Immunol. (2007) 51:993–1001. doi: 10.1111/j.1348-0421.2007.tb03996.x, PMID: 17951989

[139] Hashizume H Fujiyama T Kanebayashi J Kito Y Hata M Yagi H. Skin recruitment of monomyeloid precursors involves human herpesvirus-6 reactivation in drug allergy. Allergy. (2013) 68:681–9. doi: 10.1111/all.12138, PMID: 23573902

[140] Mardivirin L Valeyrie-Allanore L Branlant-Redon E Beneton N Jidar K Barbaud A. Amoxicillin-induced flare in patients with DRESS (drug reaction with eosinophilia and systemic symptoms): report of seven cases and demonstration of a direct effect of amoxicillin on human herpesvirus 6 replication in vitro. Eur J Dermatol. (2010) 20:68–73. doi: 10.1684/ejd.2010.0821, PMID: 19822481

[141] Mardivirin L Lacroix A Delebassée S Descamps V Ranger-Rogez S. Enhancement of human herpesvirus 6 replication using sodium valproate [article]. Virologie. (2007) 11:151–3. doi: 10.1684/vir.2011.8928, PMID: 37012835

[142] Kuntz-Simon G Obert G. Sodium valproate, an anticonvulsant drug, stimulates human cytomegalovirus replication. J Gen Virol. (1995) 76:1409–15. doi: 10.1099/0022-1317-76-6-1409 7782769

[143] Takahashi R Kano Y Yamazaki Y Kimishima M Mizukawa Y Shiohara T. Defective regulatory T cells in patients with severe drug eruptions: timing of the dysfunction is associated with the pathological phenotype and outcome. J Immunol. (2009) 182:8071–9. doi: 10.4049/jimmunol.0804002, PMID: 19494333

[144] Kano Y Inaoka M Shiohara T. Association between anticonvulsant hypersensitivity syndrome and human herpesvirus 6 reactivation and hypogammaglobulinemia. Arch Dermatol. (2004) 140:183–8. doi: 10.1001/archderm.140.2.183, PMID: 14967790

[145] Kano Y Seishima M Shiohara T. Hypogammaglobulinemia as an early sign of drug-induced hypersensitivity syndrome. J Am Acad Dermatol. (2006) 55:727–8. doi: 10.1016/j.jaad.2006.02.050, PMID: 17010766

[146] Yazicioglu M Elmas R Turgut B Genchallac T. The association between DRESS and the diminished numbers of peripheral B lymphocytes and natural killer cells. Pediatr Allergy Immunol. (2012) 23:289–96. doi: 10.1111/j.1399-3038.2012.01268.x, PMID: 22432939

[147] Sugita K Tohyama M Watanabe H Otsuka A Nakajima S Iijima M. Fluctuation of blood and skin plasmacytoid dendritic cells in drug-induced hypersensitivity syndrome. J Allergy Clin Immunol. (2010) 126:408–10. doi: 10.1016/j.jaci.2010.06.004, PMID: 20624646

[148] Siegal FP Kadowaki N Shodell M Fitzgerald-Bocarsly PA Shah K Ho S. The nature of the principal type 1 interferon-producing cells in human blood. Science. (1999) 284:1835–7. doi: 10.1126/science.284.5421.1835, PMID: 10364556

[149] Colonna M Trinchieri G Liu YJ. Plasmacytoid dendritic cells in immunity. Nat Immunol. (2004) 5:1219–26. doi: 10.1038/ni1141 15549123

[150] Hsu SH Yang CW Hsieh YC Chen KL Cho YT Liau JY. Plasmacytoid dendritic cells diminution in peripheral blood is prevalent in drug reaction with eosinophilia and systemic symptoms and may precede human herpesvirus 6 reactivation [article]. Dermatol Sin. (2021) 39:175–81. doi: 10.4103/ds.ds_37_21

[151] Pichler WJ Bruggen MC. Viral infections and drug hypersensitivity. Allergy. (2022) 78:60–70. doi: 10.1111/all.15558 36264263

[152] Pichler WJ. The important role of non-covalent drug-protein interactions in drug hypersensitivity reactions. Allergy. (2022) 77:404–15. doi: 10.1111/all.14962, PMID: 34037262 PMC9291849

[153] Kano Y Hiraharas K Sakuma K Shiohara T. Several herpesviruses can reactivate in a severe drug-induced multiorgan reaction in the same sequential order as in graft-versus-host disease. Br J Dermatol. (2006) 155:301–6. doi: 10.1111/j.1365-2133.2006.07238.x, PMID: 16882166

[154] Lee C-H Huang Y-H Chu C-Y. Taiwan dermatological association recommendations for coronavirus disease of 2019 vaccination in patients treated with immunotherapeutics [review article]. Dermatol Sin. (2021) 39:169–74. doi: 10.4103/ds.ds_50_21

[155] Luo C Geng C-Z Tung Y-H Wang B-L Tung T-H. Coronavirus disease 2019 in dermatology practice: perspective of three levels of prevention on public health [review article]. Dermatol Sin. (2022) 40:143–7. doi: 10.4103/ds.ds_33_22

[156] Mitamura Y Schulz D Oro S Li N Kolm I Lang C. Cutaneous and systemic hyperinflammation drives maculopapular drug exanthema in severely ill COVID-19 patients. Allergy. (2022) 77:595–608. doi: 10.1111/all.14983, PMID: 34157151 PMC8441838

[157] Yu C-L Lin Y-T Chi C-C. Recommendations on use of systemic treatments for immune-mediated dermatologic disorders in patients with confirmed COVID-19 infection: A rapid review [review article]. Dermatol Sin. (2022) 40:67–70. doi: 10.4103/1027-8117.349030

[158] Wu PC Huang IH Wang CW Chung WH Chen CB. Severe cutaneous adverse reactions after COVID-19 vaccination: A systematic review. Allergy. (2023) 78:1383–6. doi: 10.1111/all.15642, PMID: 36627233

[159] O'Connor T O'Callaghan-Maher M Ryan P Gibson G. Drug reaction with eosinophilia and systemic symptoms syndrome following vaccination with the AstraZeneca COVID-19 vaccine. JAAD Case Rep. 20:14–6. doi: 10.1016/j.jdcr.2021.11.028 PMC8673947 34931172

[160] Schroeder JW Gamba C Toniato A Rongioletti F. A definite case of drug reaction with eosinophilia and systemic symptoms (DRESS) induced by administration of the Pfizer/BioNTech BNT162b2 vaccine for SARS-CoV2. Clin Dermatol. (2022) 40:591–4. doi: 10.1016/j.clindermatol.2022.02.018, PMID: 35550918 PMC9085440

[161] Saber M Faghihi G Seyedghafouri SA Hosseini SM. Mortality and cause of death in patients with dermatologic diseases: an 11-year record-based observational study. Dermatol Sin. (2023) 41:18. doi: 10.4103/ds.DS-D-22-00134

[162] Um SJ Lee SK Kim YH Kim KH Son CH Roh MS. Clinical features of drug-induced hypersensitivity syndrome in 38 patients. J Investig Allergol Clin Immunol. (2010) 20:556–62. PMID: 21313995

[163] Cabañas R Calderon O Ramirez E Fiandor A Prior N Caballero T. Piperacillin-induced DRESS: distinguishing features observed in a clinical and allergy study of 8 patients. J Investig Allergol Clin Immunol. (2014) 24:425–30. PMID: 25668894

[164] Momen SE Diaz-Cano S Walsh S Creamer D. Discriminating minor and major forms of drug reaction with eosinophilia and systemic symptoms: facial edema aligns to the severe phenotype. J Am Acad Dermatol. (2021) 85:645–52. Conflicts of interest None disclosed. doi: 10.1016/j.jaad.2021.04.020, PMID: 33872719

[165] Tetart F Picard D Janela B Joly P Musette P. Prolonged evolution of drug reaction with eosinophilia and systemic symptoms: clinical, Virologic, and biological features. JAMA Dermatol. (2014) 150:206–7. doi: 10.1001/jamadermatol.2013.6698, PMID: 24369386

[166] Tohyama M Hashimoto K Yasukawa M Kimura H Horikawa T Nakajima K. Association of human herpesvirus 6 reactivation with the flaring and severity of drug-induced hypersensitivity syndrome. Br J Dermatol. (2007) 157:934–40. doi: 10.1111/j.1365-2133.2007.08167.x, PMID: 17854362

[167] Wei CH Chung-Yee Hui R Chang CJ Ho HC Yang CH Lin YJ. Identifying prognostic factors for drug rash with eosinophilia and systemic symptoms (DRESS). Eur J Dermatol. (2011) 21:930–7. doi: 10.1684/ejd.2011.1550, PMID: 21951554

[168] Mizukawa Y Hirahara K Kano Y Shiohara T. Drug-induced hypersensitivity syndrome/drug reaction with eosinophilia and systemic symptoms severity score: A useful tool for assessing disease severity and predicting fatal cytomegalovirus disease. J Am Acad Dermatol. (2019) 80:670–678.e2. doi: 10.1016/j.jaad.2018.08.052, PMID: 30240780

[169] Sharma AN Murphy K Shwe S Miller M Mesinkovska NA Rojek NW. Predicting DRESS syndrome recurrence—the ReDRESS score. JAMA Dermatol. (2022) 158:1445–7. doi: 10.1001/jamadermatol.2022.3491, PMID: 36260295 PMC9582960

[170] Komatsu-Fujii T Chinuki Y Niihara H Hayashida K Ohta M Okazaki R. The thymus and activation-regulated chemokine (TARC) level in serum at an early stage of a drug eruption is a prognostic biomarker of severity of systemic inflammation. Allergol Int. (2018) 67:90–5. doi: 10.1016/j.alit.2017.06.001, PMID: 28648978

[171] Taylor L Schwarz H. Identification of a soluble OX40 isoform: development of a specific and quantitative immunoassay. J Immunol Methods. (2001) 255:67–72. doi: 10.1016/S0022-1759(01)00424-0, PMID: 11470287

[172] Wongkitisophon P Chanprapaph K Rattanakaemakorn P Vachiramon V. Six-year retrospective review of drug reaction with eosinophilia and systemic symptoms. Acta Derm Venereol. (2012) 92:200–5. doi: 10.2340/00015555-1222, PMID: 22002792

[173] Lee HY. Drug reaction with eosinophilia and systemic symptoms (DRESS). Uptodate. (2023). Retrieved June 17, 2023, from https://www.uptodate.com/contents/drug-reaction-with-eosinophilia-and-systemic-symptoms-dress

[174] Fernandez-Juarez G Perez JV Caravaca-Fontán F Quintana L Shabaka A Rodriguez E. Duration of treatment with corticosteroids and recovery of kidney function in acute interstitial nephritis. Clin J Am Soc Nephrol. (2018) 13:1851–8. doi: 10.2215/CJN.01390118, PMID: 30397027 PMC6302327

[175] Hu PF Wang PQ Chen H Hu XF Xie QP Shi J. Beneficial effect of corticosteroids for patients with severe drug-induced liver injury. J Dig Dis. (2016) 17:618–27. doi: 10.1111/1751-2980.12383, PMID: 27426618

[176] Funck-Brentano E Duong TA Bouvresse S Bagot M Wolkenstein P Roujeau JC. Therapeutic management of DRESS: a retrospective study of 38 cases. J Am Acad Dermatol. (2015) 72:246–52. doi: 10.1016/j.jaad.2014.10.032, PMID: 25592341

[177] Uhara H Saiki M Kawachi S Ashida A Oguchi S Okuyama R. Clinical course of drug-induced hypersensitivity syndrome treated without systemic corticosteroids. J Eur Acad Dermatol Venereol. (2013) 27:722–6. doi: 10.1111/j.1468-3083.2012.04547.x 22540194

[178] Zhang ZX Yang BQ Yang Q Wu M Wang GJ. Treatment of drug-induced hypersensitivity syndrome with cyclosporine. Indian J Dermatol Venereol Leprol. (2017) 83:713–7. doi: 10.4103/ijdvl.IJDVL_1084_16 28984626

[179] Harman KE Morris SD Higgins EM. Persistent anticonvulsant hypersensitivity syndrome responding to ciclosporin. Clin Exp Dermatol. (2003) 28:364–5. doi: 10.1046/j.1365-2230.2003.01267.x, PMID: 12823292

[180] Kuschel SL Reedy MS. Cyclosporine treatment of drug reaction with eosinophilia and systemic symptoms (DRESS) syndrome: a case report and brief review of the literature. Pract Dermatol. (2018) 2018:41–3. PMID: 30574026 PMC6298437

[181] Kirchhof MG Wong A Dutz JP. Cyclosporine treatment of drug-induced hypersensitivity syndrome. JAMA Dermatol. (2016) 152:1254–7. doi: 10.1001/jamadermatol.2016.2220 27438540

[182] Su HJ Chen CB Yeh TY Chung WH. Successful treatment of corticosteroid-dependent drug reaction with eosinophilia and systemic symptoms with cyclosporine. Ann Allergy Asthma Immunol. (2021) 127:674–81. doi: 10.1016/j.anai.2021.08.012, PMID: 34400311

[183] Nguyen E Yanes D Imadojemu S Kroshinsky D. Evaluation of cyclosporine for the treatment of DRESS syndrome. JAMA Dermatol. (2020) 156:704–6. doi: 10.1001/jamadermatol.2020.0048, PMID: 32159726 PMC7066519

[184] Kito Y Ito T Tokura Y Hashizume H. High-dose intravenous immunoglobulin monotherapy for drug-induced hypersensitivity syndrome. Acta Derm Venereol. (2012) 92:100–1. doi: 10.2340/00015555-1168, PMID: 21681351

[185] Scheuerman O Nofech-Moses Y Rachmel A Ashkenazi S. Successful treatment of antiepileptic drug hypersensitivity syndrome with intravenous immune globulin. Pediatrics. (2001) 107:E14. doi: 10.1542/peds.107.1.e14, PMID: 11134478

[186] Santos RP Ramilo O Barton T. Nevirapine-associated rash with eosinophilia and systemic symptoms in a child with human immunodeficiency virus infection. Pediatr Infect Dis J. (2007) 26:1053–6. doi: 10.1097/INF.0b013e318125655d, PMID: 17984815

[187] Cumbo-Nacheli G Weinberger J Alkhalil M Thati N Baptist AP. Anticonvulsant hypersensitivity syndrome: is there a role for immunomodulation? Epilepsia. (2008) 49:2108–12. doi: 10.1111/j.1528-1167.2008.01720.x, PMID: 18637830

[188] Singer EM Wanat KA Rosenbach MA. A case of recalcitrant DRESS syndrome with multiple autoimmune sequelae treated with intravenous immunoglobulins. JAMA Dermatol. (2013) 149:494–5. doi: 10.1001/jamadermatol.2013.1949, PMID: 23715168

[189] Eshki M Allanore L Musette P Milpied B Grange A Guillaume JC. Twelve-year analysis of severe cases of drug reaction with eosinophilia and systemic symptoms: a cause of unpredictable multiorgan failure. Arch Dermatol. (2009) 145:67–72. doi: 10.1001/archderm.145.1.67, PMID: 19153346

[190] Marcus N Smuel K Almog M Prais D Straussberg R Landau D. Successful intravenous immunoglobulin treatment in pediatric severe DRESS syndrome. J Allergy Clin Immunol Pract. (2018) 6:1238–42. doi: 10.1016/j.jaip.2017.10.016, PMID: 29198698

[191] Joly P Janela B Tetart F Rogez S Picard D D'Incan M. Poor benefit/risk balance of intravenous immunoglobulins in DRESS. Arch Dermatol. (2012) 148:543–4. doi: 10.1001/archderm.148.4.dlt120002-c, PMID: 22508885

[192] Laban E Hainaut-Wierzbicka E Pourreau F Yacoub M Sztermer E Guillet G. Cyclophosphamide therapy for corticoresistant drug reaction with eosinophilia and systemic symptoms (DRESS) syndrome in a patient with severe kidney and eye involvement and Epstein-Barr virus reactivation. Am J Kidney Dis. (2010) 55:e11–4. doi: 10.1053/j.ajkd.2009.10.054, PMID: 20110143

[193] Esposito AJ Murphy RC Toukatly MN Amro OW Kestenbaum BR Najafian B. Acute kidney injury in allopurinol-induced DRESS syndrome: a case report of concurrent tubulointerstitial nephritis and kidney-limited necrotizing vasculitis. Clin Nephrol. (2017) 87:316–9. doi: 10.5414/CN108966, PMID: 27900940

[194] Shaughnessy KK Bouchard SM Mohr MR Herre JM Salkey KS. Minocycline-induced drug reaction with eosinophilia and systemic symptoms (DRESS) syndrome with persistent myocarditis. J Am Acad Dermatol. (2010) 62:315–8. doi: 10.1016/j.jaad.2009.05.046, PMID: 19665822

[195] Higuchi M Agatsuma T Iizima M Yamazaki Y Saita T Ichikawa T. A case of drug-induced hypersensitivity syndrome with multiple organ involvement treated with plasma exchange. Ther Apher Dial. (2005) 9:412–6. doi: 10.1111/j.1744-9987.2005.00320.x, PMID: 16202017

[196] Lo MH Huang CF Chang LS Kuo HC Chien SJ Lin IC. Drug reaction with eosinophilia and systemic symptoms syndrome associated myocarditis: a survival experience after extracorporeal membrane oxygenation support. J Clin Pharm Ther. (2013) 38:172–4. doi: 10.1111/jcpt.12025, PMID: 23173909

[197] Hagiwara H Fukushima A Iwano H Anzai T. Refractory cardiac myocarditis associated with drug rash with eosinophilia and systemic symptoms syndrome due to anti-bipolar disorder drugs: a case report. Eur Heart J Case Rep. (2018) 2:yty100. doi: 10.1093/ehjcr/yty100, PMID: 31020177 PMC6426116

[198] Gschwend A Helbling A Feldmeyer L Mani-Weber U Meincke C Heidemeyer K. Treatment with IL5-/IL-5 receptor antagonists in drug reaction with eosinophilia and systemic symptoms (DRESS). Allergo J Int. (2022):1–8. doi: 10.1007/s40629-022-00224-7, PMID: 36035809 PMC9396594

[199] Ange N Alley S Fernando SL Coyle L Yun J. Drug reaction with eosinophilia and systemic symptoms (DRESS) syndrome successfully treated with mepolizumab. J Allergy Clin Immunol Pract. (2018) 6:1059–60. doi: 10.1016/j.jaip.2017.10.020, PMID: 29133221

[200] Thein OS Sutton B Thickett DR Parekh D. Mepolizumab rescue therapy for acute pneumonitis secondary to DRESS. BMJ Case Rep. (2019) 12:231355. doi: 10.1136/bcr-2019-231355 PMC6803131 31604720

[201] Kowtoniuk R Pinninti M Tyler W Doddamani S. DRESS syndrome-associated acute necrotizing eosinophilic myocarditis with giant cells. BMJ Case Rep. (2018) 2018. doi: 10.1136/bcr-2018-226461, PMID: [Epub ahead of print]. 30301732 PMC6194447

[202] Rubin L Talmon A Ribak Y Kessler A Martin Y Haran TK. Novel targeted inhibition of the IL-5 axis for drug reaction with eosinophilia and systemic symptoms syndrome [original research]. Front Immunol. (2023) 14:1134178. doi: 10.3389/fimmu.2023.1134178, PMID: 37187735 PMC10175640

[203] Schmid-Grendelmeier P Steiger P Naegeli MC Kolm I Claudia Cécile Valérie L Maverakis E. Benralizumab for severe DRESS in two COVID-19 patients. J Allergy Clin Immunol Pract. (2021) 9:481–483.e2. doi: 10.1016/j.jaip.2020.09.039, PMID: 33039646 PMC7543785

[204] Lang CCV Schmid-Grendelmeier P Maverakis E Brüggen MC. Reply to "Benralizumab: A potential tailored treatment for life-threatening DRESS in the COVID-19 era". J Allergy Clin Immunol Pract. (2021) 9:3531–2. doi: 10.1016/j.jaip.2021.06.048, PMID: 34273580 PMC8299284

[205] Mesli F Dumont M Soria A Groh M Turpin M Voiriot G. Benralizumab: A potential tailored treatment for life-threatening DRESS in the COVID-19 era. J Allergy Clin Immunol Pract. (2021) 9:3529–3531.e1. doi: 10.1016/j.jaip.2021.06.047, PMID: 34273579 PMC8279918

[206] Park H Choi GS Lee EM. Successful treatment of Imatinib-induced DRESS syndrome using Reslizumab without cessation of Imatinib: A case report. Case Rep Oncol. (2021) 14:1548–54. doi: 10.1159/000519471, PMID: 34899250 PMC8613629

[207] Kim D Kobayashi T Voisin B Jo JH Sakamoto K Jin SP. Targeted therapy guided by single-cell transcriptomic analysis in drug-induced hypersensitivity syndrome: a case report. Nat Med. (2020) 26:236–43. doi: 10.1038/s41591-019-0733-7, PMID: 31959990 PMC7105105

[208] Damsky WE Vesely MD Lee AI Choi J Meyer AC Chen M. Drug-induced hypersensitivity syndrome with myocardial involvement treated with tofacitinib. JAAD Case Rep. (2019) 5:1018–26. doi: 10.1016/j.jdcr.2019.07.004, PMID: 31763425 PMC6864390

[209] Chowdhury M Azari BM Desai NR Ahmad T. A novel treatment for a rare cause of cardiogenic shock. JACC Case Rep. (2020) 2:1461–5. doi: 10.1016/j.jaccas.2020.02.004, PMID: 34316997 PMC8302098

[210] Morris R Kershaw NJ Babon JJ. The molecular details of cytokine signaling via the JAK/STAT pathway. Protein Sci. (2018) 27:1984–2009. doi: 10.1002/pro.3519, PMID: 30267440 PMC6237706

[211] Lé AM Torres T. OX40-OX40L inhibition for the treatment of atopic dermatitis-focus on Rocatinlimab and Amlitelimab. Pharmaceutics. (2022) 14:2753. doi: 10.3390/pharmaceutics14122753, PMID: 36559247 PMC9787630

[212] Yoshie O. CCR4 as a therapeutic target for Cancer immunotherapy. Cancers. (2021) 13:5542. doi: 10.3390/cancers13215542, PMID: 34771703 PMC8583476

