# Supplementary material for: Advances in understanding of the pathogenesis and therapeutic implications of drug reaction with eosinophilia and systemic symptoms: an updated review
Source: Front Med (Lausanne). 2023 Jun 29;10:1187937. doi: 10.3389/fmed.2023.1187937 (PMC10338933; doi:10.3389/fmed.2023.1187937)
Supplement: Supplementary file 1 [file Table_1.docx]

**Supplementary Table S1.** Summary of characteristics of studies reporting different systemic therapeutic modalities other than corticosteroids

| **Study** | **Management** | **Patient characteristics** | **Outcome** | **Study type** | **Indication** |
| --- | --- | --- | --- | --- | --- |
| Cyclosporin | | | | | |
| Harman et al. 2003 (1) | Prednisolone 40mg/day and followed by cyclosporin 4mg/kg/day for 6 months | 1 patient | Complete resolution without recurrence | Case report | Recurrent relapsing DRESS^†^ |
| Kirchhof et al. 2016 (2) | Cyclosporin 100mg twice daily for 7 days or 5 mg/kg/day for 3 days | 2 patients | Complete resolution without recurrence | Case series | As first-line therapy |
| Zhang et al. 2017 (3) | Cyclosporin 5 mg/kg/day for 7 days, followed by 100 mg twice daily for 14 days and then 150 mg daily for 20 days | 1 patient | Complete resolution without recurrence | Case report | Recurrent relapsing DRESS with  condition contraindicated to corticosteroids (diabetes ketoacidosis) |
| Kuschel et al. 2018 (4) | Prednisolone 1mg/kg/day followed by cyclosporin 5mg/kg/day for 7 days | 1 patient | Rapid improvement | Case report | Corticosteroid-refractory DRESS |
| Nguyen et al. 2020 (5) | Cyclosporine (3-5 mg/kg/day for 7 days, tapered to 1.5-2.5 mg/kg/day for 7 days) | 5 patients treated with cyclosporin  21 patients treated with systemic steroids | Shorter time of halting progression and hospital length in cyclosporin group | Retrospective case-control | As first-line therapy |
| Su et al. 2021 (6) | Cyclosporin 0.5-3 mg/kg/day with subsequent tapering for about 18-114 days | 8 patients | Improved | Case series | Corticosteroid-refractory or recurrent relapsing DRESS |
| Intravenous immunoglobulins (IVIG) | | | | | |
| Scheuerman et al. 2001 (7) | Betamethasone 0.4 mg/kg for 5 days followed by IVIG 1g/kg/day for 2 days | 1 pediatric patient | Rapid improvement without recurrence | Case report | Corticosteroid-refractory DRESS |
| Santos et al. 2007 (8) | 2g/kg one dose | 1 pediatric patient (with HIV) | Rapid improvement with recurrence | Case report | Kawasaki syndrome was suspected initially |
| Cumbo-Nacheli et al. 2008 (9) | Corticosteroids followed by IVIG 2g/kg for 2 days and N-acetylcysteine | 1 patient | Rapid improvement | Case report | Recurrent and relapsing DRESS with life-threatening organ involvement |
| Eshki et al. 2009 (10) | Systemic steroids followed by IVIG (dose is not specified) | 3 in 15 patients (1 pediatric case) | Mortality in 2 cases  Improved in 1 case | Case series | Severe DRESS with life-threatening organ involvement |
| Joly et al. 2012 (11) | 0.2 g/kg/day for 5 days | 6 patients | Only 1 patient had complete remission^¶^ | Prospective case series | Severe DRESS (as monotherapy) |
| Kito et al. 2012 (12) | 0.4 g/kg/day for 5 days | 1 patient | Rapid improvement | Case report | Condition contraindicated to corticosteroids (pre-existing infection) (as monotherapy) |
| Singer et al. 2013 (13) | 0.5 g/kg/day every month for 8 months | 1 patient | Complete remission with autoimmune sequelae^§^ | Case report | Recurrent and relapsing DRESS |
| Marcus et al. 2018 (14) | 1-2 g/kg/day for a median time of 2 days (combined with or after use of corticosteroids) | 7 pediatric patients | Rapid improvement | Case series | Severe DRESS as initial therapy (2 patients) or add-on therapy (5 patients) |
| Cyclophosphamide | | | | | |
| Laban et al. 2010 (15) | 1 intravenous pulse of cyclophosphamide (750 mg/m^2^), relayed by oral cyclophosphamide (100 mg/day) for 6 months | 1 patient | Complete remission | Case report | Corticosteroid-refractory DRESS with severe ocular and renal involvement (require hemodialysis) |
| Esposito et al. 2017 (16) | Not specified | 1 patient | Improved | Case report | DRESS with severe renal involvement (require hemodialysis) |
| Others: Plasmapheresis, rituximab, mycophenolate mofetil (MMF) | | | | | |
| Shaughnessy et al. 2010 (17) | High dose corticosteroids + MMF + Plasmapheresis for 4 days +  Rituximab for one month | 1 patient | Improved | Case report | Recurrent relapsing DRESS with persistent myocarditis |
| Higuchi et al. 2005 (18) | Steroids pulse therapy with subsequent high dose steroids + Plasmapheresis for 4 sessions | 1 patient | Improved | Case report | Corticosteroid-refractory DRESS with severe renal involvement (require hemodialysis) |
| Lo et al. 2013 (19) | Steroids pulse therapy + ECMO + Plasmapheresis for 4 sessions | 1 patient | Improved | Case report | Corticosteroid-refractory DRESS with fulminant myocarditis |
| Hagiwara et al. 2018 (20) | High dose corticosteroids + MMF | 1 patient | Improved but not completely remitted and eventually fatal | Case report | Corticosteroid-refractory DRESS with myocarditis |
| Mepolizumab | | | | | |
| Kowtoniuk et al. 2018 (21) | Corticosteroids + MMF + ciclosporin + mepolizumab 300 mg iv monthly for 3 months followed by 500mg monthly | 1 patient | Remission without recurrence | Case report | Recurrent and relapsing DRESS with myocarditis |
| Ange et al. 2018 (22) | High dose corticosteroids + IVIG + Mepolizumab 100mg SC monthly for 3 months | 1 patient (after allogenic stem cell transplant for AML) | Remission without recurrence | Case report | Recurrent and relapsing DRESS |
| Thein et al. 2019 (23) | High dose corticosteroids + Mepolizumab 600mg SC (split to 300mg for 2 days) initially and followed by 300 mg monthly | 1 patient | Improved | Case report | Corticosteroid-refractory DRESS |
| Gschwend et al. 2022 (24) | Corticosteroids ± IVIG + Mepolizumab 100mg (total 3 doses for one case and 1 dose for another case) | 2 patients | Improved | Case report | Corticosteroid-resistant DRESS |
| Rubin et al. 2023 (25) | High dose corticosteroids + Mepolizumab 300mg SC | 1 patient | Complete remission without recurrence | Case report | Corticosteroids-dependent DRESS |
| Benralizumab | | | | | |
| Gschwend et al. 2022 (24) | Corticosteroids + Benralizumab 30mg monthly for 3 months | 1 patient | Improved | Case report | Corticosteroid-refractory DRESS (persisted hepatitis) |
| Schmid-Grendelmeier et al. 2021 (26) | Benralizumab 30mg SC | 2 patients (COVID-19 with respiratory failure) | Rapid improvement (one died of COVID) | Case report | Corticosteroid-refractory DRESS |
| Mesli et al. 2021 (27) | Corticosteroids pulse therapy + IVIG + Benralizumab 30 mg SC monthly (total 2 doses) | 1 patient (COVID-19 with respiratory failure) | Rapid improvement | Case report | Corticosteroid-refractory DRESS |
| Lang et al. 2021 (28) | Corticosteorids + Benralizumab 30 mg SC | 2 patients | Rapid improvement | Case report | Corticosteroid-refractory DRESS |
|  | Corticosteroids + Benralizumab 30 mg SC + Mepolizumab 100 mg monthly for 2 months | 1 patient | Complete remission without recurrence | Case report | Recurrent and relapsing DRESS |
| Rubin et al. 2023 (25) | Benralizumab 30 mg SC + corticosteroids | 1 patient | Complete remission without recurrence | Case report | As first-line therapy to minimize steroids toxicity |
| Reslizumab | | | | | |
| Park et al. 2021 (29) | Corticosteroids + Reslizumab 100 mg iv once and 200 mg iv once | 1 patient (imatinib-induced) | Improved without recurrence | Case report | For continued use of the culprit drug |
| Tofacitinib | | | | | |
| Damsky et al. 2019 (30) | Corticosteroids ± IVIG ± Cyclosporin ± methotrexate + Tofacitinib 5 mg twice daily | 2 patients with myocardial involvement | Improved but relapsed when tofacitinib was stopped | Case report | Recurrent, relapsing, and life-threatening DRESS |
| Kim et al. 2020 (31) | Corticosteroids + Etanercept + IVIG + Cyclosporin + MMF + Tofacitinib 5-10 mg/day for more than 10 months | 1 patient | Improved | Case report | Corticosteroid-refractory DRESS |
| Chowdhury et al. 2020 (32) | Corticosteroids + Cyclosporin + Tofacitinib 10mg twice daily | 1 patient with myocardial involvement | Rapid improvement and remission but recur when tofacitinib was stopped | Case report | Recurrent, relapsing, and life-threatening DRESS |

^†^Patient’s symptoms relapsed or deteriorated when corticosteroids are tapered or suddenly stopped.

^¶^The study was prematurely stopped for safety reason. (5 of 6 patients experienced severe adverse events and 4 patients needed to be treated with corticosteroids for the adverse events)

^§^Hypothyroidism and type I diabetes mellitus

**References**

1. Harman KE, Morris SD, Higgins EM. Persistent anticonvulsant hypersensitivity syndrome responding to ciclosporin. Clin Exp Dermatol. 2003 Jul;28(4):364-5. eng. doi:10.1046/j.1365-2230.2003.01267.x. Cited in: Pubmed; PMID 12823292.

2. Kirchhof MG, Wong A, Dutz JP. Cyclosporine Treatment of Drug-Induced Hypersensitivity Syndrome. JAMA Dermatol. 2016 Nov 1;152(11):1254-1257. eng. doi:10.1001/jamadermatol.2016.2220. Cited in: Pubmed; PMID 27438540.

3. Zhang ZX, Yang BQ, Yang Q, Wu M, Wang GJ. Treatment of drug-induced hypersensitivity syndrome with cyclosporine. Indian J Dermatol Venereol Leprol. 2017 Nov-Dec;83(6):713-717. eng. doi:10.4103/ijdvl.IJDVL_1084_16. Cited in: Pubmed; PMID 28984626.

4. Kuschel SL, Reedy MS. Cyclosporine treatment of drug reaction with eosinophilia and systemic symptoms (DRESS) syndrome: a case report and brief review of the literature. Pract Dermatol. 2018 Oct;2018:41-43. eng. The authors have no conflicts of interest relevant to this article to disclose. Cited in: Pubmed; PMID 30574026.

5. Nguyen E, Yanes D, Imadojemu S, Kroshinsky D. Evaluation of Cyclosporine for the Treatment of DRESS Syndrome. JAMA Dermatol. 2020 Jun 1;156(6):704-706. eng. Conflict of Interest Disclosures: None reported. doi:10.1001/jamadermatol.2020.0048. Cited in: Pubmed; PMID 32159726.

6. Su HJ, Chen CB, Yeh TY, Chung WH. Successful treatment of corticosteroid-dependent drug reaction with eosinophilia and systemic symptoms with cyclosporine. Ann Allergy Asthma Immunol. 2021 Dec;127(6):674-681. Epub 20210813. doi:10.1016/j.anai.2021.08.012. Cited in: Pubmed; PMID 34400311.

7. Scheuerman O, Nofech-Moses Y, Rachmel A, Ashkenazi S. Successful treatment of antiepileptic drug hypersensitivity syndrome with intravenous immune globulin. Pediatrics. 2001 Jan;107(1):E14. eng. doi:10.1542/peds.107.1.e14. Cited in: Pubmed; PMID 11134478.

8. Santos RP, Ramilo O, Barton T. Nevirapine-associated rash with eosinophilia and systemic symptoms in a child with human immunodeficiency virus infection. Pediatr Infect Dis J. 2007 Nov;26(11):1053-6. eng. doi:10.1097/INF.0b013e318125655d. Cited in: Pubmed; PMID 17984815.

9. Cumbo-Nacheli G, Weinberger J, Alkhalil M, Thati N, Baptist AP. Anticonvulsant hypersensitivity syndrome: is there a role for immunomodulation? Epilepsia. 2008 Dec;49(12):2108-12. Epub 20080714. doi:10.1111/j.1528-1167.2008.01720.x. Cited in: Pubmed; PMID 18637830.

10. Eshki M, Allanore L, Musette P, Milpied B, Grange A, Guillaume JC, Chosidow O, Guillot I, Paradis V, Joly P, Crickx B, Ranger-Rogez S, Descamps V. Twelve-year analysis of severe cases of drug reaction with eosinophilia and systemic symptoms: a cause of unpredictable multiorgan failure. Arch Dermatol. 2009 Jan;145(1):67-72. eng. doi:10.1001/archderm.145.1.67. Cited in: Pubmed; PMID 19153346.

11. Joly P, Janela B, Tetart F, Rogez S, Picard D, D'Incan M, Descamps V, Collet E, Roujeau JC, Musette P. Poor benefit/risk balance of intravenous immunoglobulins in DRESS. Arch Dermatol. 2012 Apr;148(4):543-4. eng. doi:10.1001/archderm.148.4.dlt120002-c. Cited in: Pubmed; PMID 22508885.

12. Kito Y, Ito T, Tokura Y, Hashizume H. High-dose intravenous immunoglobulin monotherapy for drug-induced hypersensitivity syndrome. Acta Derm Venereol. 2012 Jan;92(1):100-1. eng. doi:10.2340/00015555-1168. Cited in: Pubmed; PMID 21681351.

13. Singer EM, Wanat KA, Rosenbach MA. A case of recalcitrant DRESS syndrome with multiple autoimmune sequelae treated with intravenous immunoglobulins. JAMA Dermatol. 2013 Apr;149(4):494-5. eng. doi:10.1001/jamadermatol.2013.1949. Cited in: Pubmed; PMID 23715168.

14. Marcus N, Smuel K, Almog M, Prais D, Straussberg R, Landau D, Scheuerman O. Successful Intravenous Immunoglobulin Treatment in Pediatric Severe DRESS Syndrome. J Allergy Clin Immunol Pract. 2018 Jul-Aug;6(4):1238-1242. eng. Epub 20171201. doi:10.1016/j.jaip.2017.10.016. Cited in: Pubmed; PMID 29198698.

15. Laban E, Hainaut-Wierzbicka E, Pourreau F, Yacoub M, Sztermer E, Guillet G, Touchard G, Bridoux F. Cyclophosphamide therapy for corticoresistant drug reaction with eosinophilia and systemic symptoms (DRESS) syndrome in a patient with severe kidney and eye involvement and Epstein-Barr virus reactivation. Am J Kidney Dis. 2010 Mar;55(3):e11-4. eng. Epub 20100127. doi:10.1053/j.ajkd.2009.10.054. Cited in: Pubmed; PMID 20110143.

16. Esposito AJ, Murphy RC, Toukatly MN, Amro OW, Kestenbaum BR, Najafian B. Acute kidney injury in allopurinol-induced DRESS syndrome: a case report of concurrent tubulointerstitial nephritis and kidney-limited necrotizing vasculitis . Clin Nephrol. 2017 Jun;87(6):316-319. eng. doi:10.5414/cn108966. Cited in: Pubmed; PMID 27900940.

17. Shaughnessy KK, Bouchard SM, Mohr MR, Herre JM, Salkey KS. Minocycline-induced drug reaction with eosinophilia and systemic symptoms (DRESS) syndrome with persistent myocarditis. J Am Acad Dermatol. 2010 Feb;62(2):315-8. eng. Epub 20090808. doi:10.1016/j.jaad.2009.05.046. Cited in: Pubmed; PMID 19665822.

18. Higuchi M, Agatsuma T, Iizima M, Yamazaki Y, Saita T, Ichikawa T, Kamijo Y, Arakura H, Hora K, Kiyosawa K. A case of drug-induced hypersensitivity syndrome with multiple organ involvement treated with plasma exchange. Ther Apher Dial. 2005 Oct;9(5):412-6. eng. doi:10.1111/j.1744-9987.2005.00320.x. Cited in: Pubmed; PMID 16202017.

19. Lo MH, Huang CF, Chang LS, Kuo HC, Chien SJ, Lin IC, Lin KM, Lin YJ. Drug reaction with eosinophilia and systemic symptoms syndrome associated myocarditis: a survival experience after extracorporeal membrane oxygenation support. J Clin Pharm Ther. 2013 Apr;38(2):172-4. eng. Epub 20121122. doi:10.1111/jcpt.12025. Cited in: Pubmed; PMID 23173909.

20. Hagiwara H, Fukushima A, Iwano H, Anzai T. Refractory cardiac myocarditis associated with drug rash with eosinophilia and systemic symptoms syndrome due to anti-bipolar disorder drugs: a case report. Eur Heart J Case Rep. 2018 Dec;2(4):yty100. eng. Epub 20181010. doi:10.1093/ehjcr/yty100. Cited in: Pubmed; PMID 31020177.

21. Kowtoniuk R, Pinninti M, Tyler W, Doddamani S. DRESS syndrome-associated acute necrotizing eosinophilic myocarditis with giant cells. BMJ Case Rep. 2018 Oct 8;2018. eng. Competing interests: None declared. Epub 20181008. doi:10.1136/bcr-2018-226461. Cited in: Pubmed; PMID 30301732.

22. Ange N, Alley S, Fernando SL, Coyle L, Yun J. Drug Reaction with Eosinophilia and Systemic Symptoms (DRESS) syndrome successfully treated with mepolizumab. J Allergy Clin Immunol Pract. 2018 May - Jun;6(3):1059-1060. Epub 20171110. doi:10.1016/j.jaip.2017.10.020. Cited in: Pubmed; PMID 29133221.

23. Thein OS, Sutton B, Thickett DR, Parekh D. Mepolizumab rescue therapy for acute pneumonitis secondary to DRESS. BMJ Case Rep. 2019 Oct 10;12(10). Competing interests: None declared. Epub 20191010. doi:10.1136/bcr-2019-231355. Cited in: Pubmed; PMID 31604720.

24. Gschwend A, Helbling A, Feldmeyer L, Mani-Weber U, Meincke C, Heidemeyer K, Bossart S, Jorg L. Treatment with IL5-/IL-5 receptor antagonists in drug reaction with eosinophilia and systemic symptoms (DRESS). Allergo J Int. 2022 Aug 23:1-8. Conflict of interestL. Jorg has received advisory board fees from Astra Zeneca and speaker fees from GSK. A. Helbling has received advisory board fees from Astra Zeneca and GSK. A. Gschwend has received speaker fees from GSK. L. Feldmeyer, U. Mani-Weber, C. Meincke, K. Heidemeyer and S. Bossart declare that they have no competing interests. Epub 20220823. doi:10.1007/s40629-022-00224-7. Cited in: Pubmed; PMID 36035809.

25. Rubin L, Talmon A, Ribak Y, Kessler A, Martin Y, Haran TK, Shamriz O, Adini I, Tal Y. Novel targeted inhibition of the IL-5 axis for drug reaction with eosinophilia and systemic symptoms syndrome [Original Research]. Frontiers in Immunology. 2023 2023-April-28;14. English. doi:10.3389/fimmu.2023.1134178.

26. Schmid-Grendelmeier P, Steiger P, Naegeli MC, Kolm I, Claudia Cécile Valérie L, Maverakis E, Brüggen MC. Benralizumab for severe DRESS in two COVID-19 patients. J Allergy Clin Immunol Pract. 2021 Jan;9(1):481-483.e2. eng. Epub 20201008. doi:10.1016/j.jaip.2020.09.039. Cited in: Pubmed; PMID 33039646.

27. Mesli F, Dumont M, Soria A, Groh M, Turpin M, Voiriot G, Rafat C, Sallé DS, Gibelin A, Desnos C. Benralizumab: A potential tailored treatment for life-threatening DRESS in the COVID-19 era. J Allergy Clin Immunol Pract. 2021 Sep;9(9):3529-3531.e1. eng. Epub 20210715. doi:10.1016/j.jaip.2021.06.047. Cited in: Pubmed; PMID 34273579.

28. Lang CCV, Schmid-Grendelmeier P, Maverakis E, Brüggen MC. Reply to "Benralizumab: A potential tailored treatment for life-threatening DRESS in the COVID-19 era". J Allergy Clin Immunol Pract. 2021 Sep;9(9):3531-3532. eng. Epub 20210715. doi:10.1016/j.jaip.2021.06.048. Cited in: Pubmed; PMID 34273580.

29. Park H, Choi GS, Lee EM. Successful Treatment of Imatinib-Induced DRESS Syndrome Using Reslizumab without Cessation of Imatinib: A Case Report. Case Rep Oncol. 2021 Sep-Dec;14(3):1548-1554. eng. The authors have no conflicts of interest to declare. Epub 20211022. doi:10.1159/000519471. Cited in: Pubmed; PMID 34899250.

30. Damsky WE, Vesely MD, Lee AI, Choi J, Meyer AC, Chen M, Ahmad T, King B. Drug-induced hypersensitivity syndrome with myocardial involvement treated with tofacitinib. JAAD Case Rep. 2019 Dec;5(12):1018-1026. Epub 20191113. doi:10.1016/j.jdcr.2019.07.004. Cited in: Pubmed; PMID 31763425.

31. Kim D, Kobayashi T, Voisin B, Jo JH, Sakamoto K, Jin SP, Kelly M, Pasieka HB, Naff JL, Meyerle JH, Ikpeama ID, Fahle GA, Davis FP, Rosenzweig SD, Alejo JC, Pittaluga S, Kong HH, Freeman AF, Nagao K. Targeted therapy guided by single-cell transcriptomic analysis in drug-induced hypersensitivity syndrome: a case report. Nat Med. 2020 Feb;26(2):236-243. Epub 20200120. doi:10.1038/s41591-019-0733-7. Cited in: Pubmed; PMID 31959990.

32. Chowdhury M, Azari BM, Desai NR, Ahmad T. A Novel Treatment for a Rare Cause of Cardiogenic Shock. JACC Case Rep. 2020 Aug;2(10):1461-1465. Epub 20200422. doi:10.1016/j.jaccas.2020.02.004. Cited in: Pubmed; PMID 34316997.
